# Selection for Short-Nose and Small Size Creates a Behavioural Trade-Off in Dogs

**DOI:** 10.3390/ani15152221

**Published:** 2025-07-28

**Authors:** Borbála Turcsán, Eniko Kubinyi

**Affiliations:** 1 Department of Ethology, ELTE Eötvös Loránd University, 1117 Budapest, Hungary; eniko.kubinyi@ttk.elte.hu; 2 MTA-ELTE Lendület “Momentum” Companion Animal Research Group, 1117 Budapest, Hungary

**Keywords:** dog, head shape, management characteristics, body size, behaviour, welfare

## Abstract

Brachycephalic head shape in dogs has been associated with behavioural traits that may enhance their appeal as companion animals, contributing to their popularity. However, it remains unclear whether these behavioural differences are directly linked to head shape or are mediated by factors such as body size, demographics, and dog-keeping practices. Drawing on two large-scale owner surveys (N = 5613) and cephalic index estimates for 90 breeds, we investigated the relationship between head shape and eight behavioural variables (four personality traits and four behavioural problems), while controlling for 20 demographic and dog-keeping characteristics, as well as body size. Our results show that behavioural differences among head shapes are only partly attributable to head shape itself; some are explained by confounding variables. Specifically, brachycephalic dogs appeared predisposed to positive behaviours (e.g., calmness, fewer behavioural problems), but these traits were often obscured by their small body size and low training experience. These findings highlight the complex interplay between morphology, behaviour, and environment, and emphasize the role of training and management in supporting the behavioural well-being of popular brachycephalic breeds. This has important implications for owners, breeders, and welfare professionals aiming to align aesthetic preferences with behavioural and welfare outcomes.

## Simple Summary

The popularity of flat-faced (brachycephalic) dog breeds, such as French Bulldogs and Pugs, is steadily rising, despite growing concerns about their health and welfare. One possible reason for this trend is that these dogs exhibit behavioural traits that make them especially good companions. However, it is unclear whether this perception is accurate and, if so, whether these traits are truly linked to their head shape. In this study, we analyzed data from over 5000 dogs across 90 breeds to explore how head shape relates to behaviour, while also taking into account body size, owner demographics, and dog-keeping practices. The initial results did not support that brachycephalic dogs display more positive behavioural traits. However, specific statistical methods revealed that some of their negative characteristics, like their reduced trainability and higher reactivity, are mainly due to their small size, limited training experience, and their owners’ spoiling behaviour rather than head shape itself. At the same time, brachycephalic dogs also have some positive traits linked to their head shape, such as lower arousal, but these often go unnoticed in everyday life due to the same confounding factors. Moreover, the flat-faced dogs’ higher calmness, boldness, and lower obedience when called seem unrelated to body size, environment, or keeping practices, suggesting direct links between skull morphology, genetics, and behaviour. This research helps explain why brachycephalic breeds are so appealing to many owners, and highlights the importance of proper training and care in supporting their well-being.

## 1. Introduction

The popularity of brachycephalic (short-nosed) dogs is steadily growing worldwide [1,2], despite serious health, welfare, and longevity issues, including upper respiratory diseases (e.g., brachycephalic obstructive airway syndrome), allergies, and corneal ulceration [3,4]. Owners of these breeds are also highly likely to remain loyal to their breed (93.0%) and recommend them to others (65.5%) [5]. This so-called “brachycephalic paradox” [6] has motivated researchers to explore the appeal of these dogs.

According to the most widely accepted explanation, short-nosed dogs’ popularity is driven by the “baby schema effect”, which suggests that paedomorphic facial features, such as large heads, round faces, large eyes, and small noses (which are characteristic features of brachycephalic dogs [7]), trigger the “cute response,” eliciting attraction, positive emotions, and protective and caregiving behaviours in parents and non-parents alike [8,9,10,11,12]. Supporting this idea, appearance is reportedly more important to owners of brachycephalic dogs compared to owners of other breeds [5,13], and owners of short-nosed dogs tend to form stronger bonds with their pets [4], likely due to their higher perceived need for care [14].

Alternative, though not mutually exclusive, explanations have also been proposed, including fashion trends and social contagion, way of self-expression, status symbolism, and the desire for social acknowledgment [14,15].

Recent studies have proposed that, aside from their appearance, these dogs also exhibit infant-like behavioural traits or other desirable behaviours that might outweigh concerns about their health and welfare problems [5,16,17]. For instance, qualitative data from owners of brachycephalic dogs suggest that these breeds display behavioural traits well suited for companionship, a sedentary lifestyle, and compatibility with children [5].

Several empirical studies also indicate that brachycephalic breeds exhibit distinctive behavioural traits that may enhance their suitability as companion animals. They are frequently described as affectionate, playful, interactive, and highly sociable with humans [18,19]. Research suggests that brachycephalic dogs demonstrate superior visual communicative abilities, establishing eye contact with humans more readily [16], following human pointing gestures more successfully [20], and looking longer at human and dog portraits compared to longer-nosed breeds [21]. Additionally, they tend to orient toward humans rather than independently solving problems, a trait that may be perceived as dependence or helplessness by owners [17]. However, despite these positive social attributes, brachycephalic breeds have also been associated with behavioural challenges such as higher dog-directed aggression [18] and lower trainability [22].

These findings suggest that head shape may influence behavioural tendencies, affecting both personality traits and the prevalence of behavioural problems. One possible explanation involves the neural architecture of brachycephalic breeds: as skull morphology and brain morphology covary, specific skull shapes are known to be associated with structural changes or reductions in particular brain regions, which may in turn have profound effects on behaviour [23]. For example, differences in retinal ganglion cell distribution might contribute to their enhanced visual communication abilities [24]. However, recent research suggests that their tendency to form eye contact does not directly contribute to their popularity [6]. Another hypothesis proposed by McGreevy et al. [18] suggests that brachycephalic head shapes may be a by-product of human selection for neotenous characteristics, whereas dolichocephalic breeds were selected for hunting and chasing abilities. Finally, these behavioural differences may be shaped by nurture rather than nature. Previous studies comparing dogs or breeds based on cephalic index or head shape did not adequately control for potential systematic differences between them in other morphological, environmental, and dog-keeping factors. For example, brachycephalic breeds are typically selected for different reasons than other breeds [5], are more likely to live in apartments [13], and tend to be kept by a specific group of owners [6,13].

This is a crucial consideration, as environmental factors, such as living conditions, life and training history, owner demographics, and attitudes, are known to be associated with behaviour and the prevalence of behavioural problems [25,26,27,28,29,30,31,32,33,34,35]. Accordingly, it is also possible that brachycephalic breeds are not genetically predisposed to behave differently than longer-nosed dogs, but they are acquired for different reasons, or kept and treated differently, by different types of owners, and the resulting differences in life experiences, training history, and living environment results in the observed differences in behaviour.

One more factor needs to be considered, namely body size. Most of the popular brachycephalic breeds (e.g., Pug, French bulldog) are small-sized, and smaller dogs tend to have more brachycephalic skull shapes [36]. Body size is an important factor that negatively affects behaviour and increases the prevalence of behavioural problems [18,19,26,32,37,38], and it is closely intertwined with the animal’s living environment [28,33,35,37,39]. Previous studies have suggested that owners may tolerate undesirable behaviours in smaller dogs because they pose less risk [40] or that the way these dogs are kept fosters fear and frustration, increasing problematic behaviours [19,37]. It is also possible that selection for small body size led to neurological changes which affect how dogs react to their environment [18]. Thus, the behavioural differences between breeds of different head shapes could simply be a by-product of the differences between small and large-sized dogs. In sum, it remains unclear to what extent associations between head shape and behaviour are genetically or environmentally determined.

This study aimed to investigate whether behavioural differences commonly attributed to head shape are genuinely caused by head shape itself, or rather the result of other morphological, environmental, or demographic factors associated with head shape. While it is possible that small-sized and/or brachycephalic dogs are genetically predisposed to display neotenous behaviours that elicit caregiving from humans [18,19], we hypothesized that behavioural differences between head shapes are, at least in part, mediated by factors such as body size, training experiences, owner characteristics, and dog-keeping practices.

Specifically, our objectives were to achieve the following:

1. Establish the baseline (unadjusted) associations between head shape and behavioural variables.
2. Identify potential confounding factors that are associated with both head shape and behaviour.
3. Evaluate whether these factors act as mediators or suppressors of the observed relationships between head shape and behaviour.
4. Replicate all analyses excluding large-sized brachycephalic dogs to test whether previously reported behavioural associations primarily apply to the more typical small-sized companion breeds within the brachycephalic group, and to assess whether size variation within this group inflates or obscures these associations.

## 2. Materials and Methods

### 2.1. Subjects

We collected responses from N = 14,004 dog owners in the first survey and N = 10,240 in the second. Among them, N = 312 owners participated in both surveys; for these cases, their demographic and dog-keeping responses were considered only once. We excluded reports with missing data, duplicate entries (i.e., cases where owners submitted multiple reports for the same dog), and reports on mixed-breed dogs.

To prevent a few highly popular breeds from disproportionately influencing group values, we capped the number of individuals per breed at 100. If a breed exceeded this threshold, we randomly selected 100 individuals for the final dataset.

From the remaining purebred dogs, we included only those for which breed-average cephalic index (CI) estimates were available. These CI values were derived from the merged dataset of Stone et al. [19] and our own dataset [16] (see details in [41]). In the final dataset, CI estimates were available for N = 113 breeds, with N = 4385 individuals from the first survey and N = 3578 from the second.

Since no universally accepted cut-off values exist for defining head shape categories based on the cephalic index, we established classification thresholds informed by values commonly used in the literature and typically applied in anatomical studies (e.g., [41]). Considering that even within breeds there is natural individual variation in CI, we decided to use non-contiguous categories when setting our thresholds, that is, we defined our groups by distinct, non-overlapping threshold ranges. Although this method led to the exclusion of 23 breeds from the analyses, when lacking individual head shape measurements, this method helps to reduce misclassification due to measurement error or ambiguity near category boundaries, and it also ensures clear separation without arbitrary divisions. Accordingly, breeds with a CI below 50 were classified as dolichocephalic (N = 1439 individuals, 28 breeds), those with a CI between 53 and 62 as mesocephalic (N = 2388, 35 breeds), and those with a CI above 65 as brachycephalic (N = 1786, 27 breeds) (Supplementary Table S1).

However, the brachycephalic group, when defined solely by CI, also includes large-bodied molossoid breeds, which likely differ from smaller, predominantly allometric brachycephalic dogs in both behaviour and keeping practices—if for no other reason than their size. To account for this potential variation and investigate whether the observed patterns were driven by the smaller breeds, we conducted our analyses in two ways: first, including all brachycephalic dogs (main text), and second, excluding large-sized (>40 cm height) brachycephalic dogs (N = 1256 individuals, 19 breeds) (Supplementary Tables S3, S4 and S6).

### 2.2. Procedure

The surveys used in this study were previously described in Turcsán et al. [42]. Briefly, two owner-report surveys were conducted in Germany: the first (Survey 1) focused on assessing dog personality, while the second (Survey 2) investigated problematic behaviours of the dogs, as perceived by their owners.

Survey 1 consisted of 24 items, 17 of which were grouped into four personality traits ([29,43]; Supplementary Table S2):

1. Calmness
2. Trainability
3. Dog sociability
4. Boldness

Survey 2 assessed 12 common behavioural problems, along with owners’ negative attitudes toward these behaviours. For each statement, owners rated their level of agreement on a 3-point scale. In the present study, we selected the four most prevalent problematic behaviours for analysis (Supplementary Table S2):

1. Jumping up on people
2. Pulling on the leash
3. Too reactive when guests arrive
4. Not coming back when called

Since the statistical package used for testing the mediation effect (lavaan [44]) did not support multinomial type as a dependent variable, we recoded responses into a binary (yes/no) format: responses of “agree” or “partly agree” were categorized as “yes”, while “disagree” was categorized as “no”. In the following, we refer to the four personality traits and the four problematic behaviours as eight “behavioural variables”.

Both surveys also collected demographic data on owners and dogs, as well as information on dog-keeping practices. Twelve questions were common to both surveys, while two questions were exclusive to Survey 1 and six to Survey 2. Additionally, we included dog weight and height data obtained from breed standards (FCI and AKC). If breed standards specified different size ranges for males and females, we calculated these separately.

Since height and weight were strongly correlated (r = 0.893, *p* < 0.001), including both in the same statistical model would lead to multicollinearity issues. To address this, we accounted for both traits by including in the model the weight and the residual of height after controlling for weight (hereafter called height residual). This measure shows how much an individual is taller or shorter than expected for their body weight, so it effectively captures the height variation that is independent of weight. The height residual was extracted from a linear regression model.

Generative AI assistance was used in the preparation of this manuscript. Specifically, ChatGPT (GPT-4-turbo, OpenAI, San Francisco, CA, USA) was employed to refine the language, grammar, and style of the text, based on author-provided content. In addition, the model was used to generate illustrative images of dog head shapes, which were created based on detailed descriptions provided by the authors. All text and images produced by the AI were critically reviewed, edited, and, where necessary, revised by the authors to ensure scientific accuracy and appropriateness. No AI tools were used to generate, analyze, or interpret the study data.

### 2.3. Statistical Analyses

**Figure 1:**
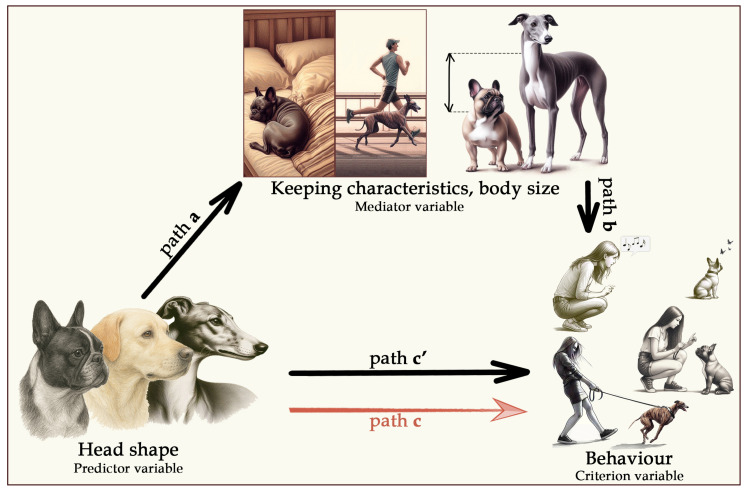
Schematic path diagram of the mediation model tested in the current study.

The data that support the findings of this study are available on figshare with the identifier doi: 10.6084/m9.figshare.28815485 [45]. All analyses were conducted at the individual level, with each dog treated as a separate data point. To determine whether potential confounders (body size, demographic factors, and dog-keeping characteristics) mediate the relationship between head shape and behaviour, we applied the four-step criteria of mediation outlined by Baron and Kenny [46] (see Figure 1). Our analysis systematically tested these criteria step by step.

1. The predictor variable (head shape) must be significantly associated with the criterion variable (behaviour and behavioural problems)—this corresponds to path c in Figure 1.
2. The predictor variable (head shape) must also correlate with the proposed mediator (body size, demographic factors, or dog-keeping characteristics)—this is path a in Figure 1.
3. The proposed mediator must, in turn, be significantly associated with the criterion variable (behaviour)—this is path b.
4. There is a mediation effect if, by including the indirect path between the predictor (head shape) and the criterion variable (behaviour) (i.e., paths a + b) in the model, the adjusted direct association (path c’) becomes weaker than the initial association (path c).

In addition to this traditional mediation framework, we also tested for a suppression effect, where an initially non-significant relationship between the predictor (head shape) and the criterion variable (behaviour) becomes significant after controlling for a confounding factor. In this case, the first and fourth criteria of the mediation model are reversed: the predictor and criterion are not significantly associated at first (path c), but a significant relationship emerges once the confounding factor is accounted for (path c’).

#### 2.3.1. Baseline Behavioural Differences Between Head Shape Groups (Path c)

The first set of models examined baseline behavioural differences across head shape groups. Since the assumptions of normality of residuals and homogeneity of variance were not met (based on visual inspection of the QQ plot of the residuals and by assessing the homogeneity of variance using the Levene test), we applied generalized linear models (GLMs) with robust covariance matrix estimation. The four personality traits from Survey 1 were analyzed using linear models. The four behavioural problems from Survey 2 were assessed using binomial logistic models. In all models, the head shape group was the sole fixed factor. If a significant effect was detected, we conducted pairwise post hoc comparisons to identify specific group differences. The effect sizes for these pairwise differences were estimated using the odds ratio (Exp(B)).

#### 2.3.2. Associations Between Potential Confounding Factors and Head Shape (Path a)

We next examined whether body size and dog-keeping characteristics differed across head shape groups, as these factors could potentially confound behavioural differences. For the 12 demographic and dog-keeping factors common to both surveys, we pooled the data from both sources. Categorical variables were compared using Chi-squared tests with z post hoc tests. Continuous variables (age, weight, and height residual) were analyzed using a Kruskal–Wallis test. To quantify group differences in demographic and dog-keeping factors, we reported unstandardized effect sizes, as we considered these more meaningful than standardized measures [47].

Although analyzing each factor separately increased the number of comparisons (which we accounted for by adjusting the significance threshold, see below), we did not include all factors in a single model for two reasons. First, only 14 of the 22 potential confounders were available for all dogs (12 demographic and dog-keeping characteristics and 2 body size measures). Second, the factors were not necessarily independent of each other, meaning that including them in a single model could introduce bias. Given the large number of comparisons in this and the following step (Section 2.3.3), we applied a Bonferroni correction to control for multiple testing, setting the significance threshold at *p* = 0.00037 (0.05/134 (22 + 12 × 8 + 1 × 4 + 3 × 4)). Only factors that significantly differed between head shape groups were included in further analyses.

#### 2.3.3. Associations Between Potential Confounding Factors and Behaviour (Path b)

To examine the relationship between each potential confounder and the behavioural variables, we analyzed them separately, following the same rationale as outlined in the previous section. This second set of generalized linear models (GLMs) had a similar structure to the first: the eight behavioural variables served as dependent factors, and each model included only one fixed factor (for categorical variables) or one covariate (for continuous variables), representing the specific body size, demographic, or dog-keeping characteristic under investigation.

We estimated effect sizes using odds ratios (Exp(B)). As before, only confounding factors that showed a significant association with a given behavioural variable were included in subsequent analyses of that variable.

#### 2.3.4. Behaviour Differences After Controlling for Potential Confounding Factors (Path c’)

Since multiple confounders met both the second and third mediation criteria for each behavioural variable, we first controlled for all these factors simultaneously to determine whether the initial behavioural differences (or the lack thereof) between head shape groups changed after accounting for potential confounders.

The third set of generalized linear models (GLMs) included the eight behavioural variables as dependent factors (using either a linear or binomial distribution, depending on the variable), while explanatory factors included head shape and all confounders significantly associated with each behavioural variable (age, weight, and height residual as covariates, and categorical variables as fixed factors).

In cases where controlling for confounders altered the association between head shape groups and behaviour (i.e., when a notable difference was observed between path c and path c’), we further examined whether these factors truly mediated the relationship between head shape and behaviour, or whether the observed effect of head shape was merely a by-product of these confounding factors.

To achieve this, we identified key confounding factors for each behavioural trait—factors that, when included individually, were sufficient to alter the association between head shape and behaviour. Specifically, we re-ran each behavioural model, including only head shape group and one potential confounder at a time. If the significance of the head shape group effect changed in the expected direction, that factor was classified as a key confounder and included in later mediation analyses. If no single confounder was sufficient to induce the change, we tested whether the combined effect of the strongest confounders could account for the observed pattern.

#### 2.3.5. Individual Mediation Models

To assess the mediation effect of the identified key confounder(s), we employed the R package lavaan (v.0.6) [44]. Mediation models were fitted using the ‘sem’ function, and bootstrapping (1000 repetitions) was applied to generate 95% confidence intervals for the model parameters. Mediation was considered statistically supported if the confidence interval of the mediation effect did not include zero.

When multiple key confounders were identified, each was tested individually in separate models, and subsequently included in a multi-mediation model. If no key confounders were found (i.e., the combined effect of two confounders was necessary to alter the head shape-behaviour association), we proceeded directly to multi-mediation models. In these latter models, we also added the relationships between confounders: a regression when causality was clear, and covariance estimation when it was not.

All GLM, Chi-squared, and Kruskal–Wallis analyses were conducted using IBM SPSS (version 28.0), while mediation analyses were performed in RStudio (version 2024.12.0+467) within the R statistical environment [48].

## 3. Results

### 3.1. Baseline Behavioural Differences Between Head Shape Groups

In Survey 1, dolichocephalic dogs were rated as less calm and less bold compared to both mesocephalic and brachycephalic dogs. Additionally, brachycephalic dogs were perceived as less trainable than both mesocephalic and dolichocephalic dogs. No significant differences were found in dog sociability across the three head shape groups (Table 1).

In Survey 2, brachycephalic dogs were reported to be more reactive to arriving guests compared to meso- and dolichocephalic dogs, and mesocephalic dogs were more reactive than dolichocephalic dogs. Additionally, both brachycephalic and mesocephalic dogs were less likely to return when called than dolichocephalic dogs. However, no significant differences were observed between the head shape groups in jumping up on people or pulling on the leash (Table 1).

When large-sized brachycephalic dogs were excluded, the only change compared to the above-described differences is that dolichocephalic dogs are less bold only compared to mesocephalic dogs, but not to brachycephalic dogs (Supplementary Table S3).

### 3.2. Associations Between Potential Confounding Factors and Head Shape

We identified significant differences between the head shape groups for 16 out of the 22 demographic and dog-keeping factors examined, even after correcting for multiple comparisons (Table 2). Twelve of these factors were common to both surveys, one was included only in Survey 1, and two factors were unique to Survey 2. However, despite the large sample size and the *p* < 0.00037 threshold, most of the differences had a small effect size. Among the categorical variables, only seven factors showed a >10% difference between the dog groups in any given category.

Owners of brachycephalic and mesocephalic dogs reported having less prior experience with dogs compared to owners of dolichocephalic dogs. Additionally, the owners of brachycephalic dogs were more likely to fall within the 19–30 years of age group and less likely to be in the 30–60 age range. Brachycephalic dogs were more likely to be kept solely as family companions, to receive no formal training, to be housed exclusively indoors, to have daily walks shorter than an hour, and to be allowed on the owner’s bed compared to meso- and dolichocephalic dogs. In addition to these notable differences, we also observed differences with smaller effect size, such as brachycephalic dogs being less likely to be neutered, and their owners being more likely to be women, with a college degree, living alone, and spending more than three hours a day with their dog.

Regarding continuous variables, brachycephalic dogs were slightly younger than the other groups, but notably smaller in both weight and height compared to meso- and dolichocephalic dogs. No significant differences were found between the groups in terms of the dog’s sex, the time spent playing with the dog, whether gifts were purchased for the dog, the number of other dogs, or the number of children in the household (Table 2).

When large-sized brachycephalic dogs were excluded, there were only two changes compared to the above-described differences. The difference between head shape groups in owner education was no longer significant enough to pass the Bonferroni correction, but the difference in time spent playing was: the owners spent more time playing with their small-sized brachycephalic dogs than with both other groups (Supplementary Table S4).

### 3.3. Associations Between Potential Confounding Factors and Behaviour

For statistical details of all associations between demographic and dog-keeping factors and behavioural variables, see Supplemental Tables S5 and S6. Below, we list the significant ones.

*Calmness*: Three factors had significant associations after Bonferroni correction: neutered dogs were less calm, while dogs acquired at a younger age and dogs that spent more time with their owners were calmer.

When large-sized brachycephalic dogs were excluded, aside from these, time spent with playing was also linked to calmness: more time playing was related to higher calmness.

*Trainability*: Seven factors had significant associations after Bonferroni correction: older and neutered dogs were less trainable, while dogs acquired at a younger age, dogs that spent more time with their owners, dogs that participated in more training activities, and dogs kept for reasons other than being a family member were more trainable. The height residual was positively associated with trainability.

When large-sized brachycephalic dogs were excluded, neutering and time spent together no longer reached the Bonferroni-corrected significance level, but time spent with playing did: more time playing was related to higher trainability.

*Dog sociability*: Five factors had significant associations after Bonferroni correction: older and neutered dogs were less sociable towards other dogs than younger dogs. Sociability was higher in dogs acquired between 2 and 12 weeks of age compared to those acquired at older or younger ages, in dogs that spent more time with their owners, and in dogs that participated in more training activities.

When large-sized brachycephalic dogs were excluded, aside from these, time spent with playing was also linked to calmness: more time playing was related to higher sociability towards other dogs.

*Boldness*: Three factors had significant associations after Bonferroni correction: older and neutered dogs were less bold, while dogs acquired at a younger age were bolder than dogs acquired at older ages.

Excluding large-sized brachycephalic dogs did not change the results.

*Jumping up*: Four factors had significant associations after Bonferroni correction: older dogs, neutered dogs, and dogs that participated in at least three training activities were less likely to jump up. Weight was negatively associated with the occurrence of this behaviour.

Excluding large-sized brachycephalic dogs did not change the results.

*Pulling the leash*: Five factors had significant associations after Bonferroni correction: more experienced owners, as well as owners who kept their dogs for reasons other than being a family member, reported fewer instances of leash-pulling. Older dogs, heavier dogs, and dogs that participated in at least three training activities were also less likely to exhibit this behaviour.

When large-sized brachycephalic dogs were excluded, owner experience and weight no longer reached the Bonferroni-corrected significance level.

*Too reactive when guests arrive*: Three factors had significant associations after Bonferroni correction: more experienced owners reported fewer instances of this behaviour problem, while owners who allowed their dogs into their bed reported more. Weight was negatively associated with the occurrence of this problem.

When large-sized brachycephalic dogs were excluded, owner experience no longer reached the Bonferroni-corrected significance level, but the height residual did, and it was negatively associated with this behavioural problem.

*Not coming back when called*: Five factors had significant associations after Bonferroni correction: older and more experienced owners, as well as owners who walked their dogs more, reported fewer recall problems. Similarly, older dogs and dogs that participated in at least three training activities were less likely to display recall issues.

Excluding large-sized brachycephalic dogs did not change the results.

### 3.4. Behavioural Differences After Controlling for Potential Confounding Factors

When controlling for all potential confounders significantly associated with both the head shape groups and the behaviour traits, we found that the head shape-behaviour association changed at least partially for five of the eight behavioural variables (Table 3). We also identified if there were key confounder(s) for each behaviour variable (i.e., factor(s) that alone could alter the association between head shape and behaviour).

In *trainability*, the differences between brachycephalic dogs and the other two groups (*p* < 0.001 for both) were no longer significant after controlling for the confounders (*p* > 0.3 for both). The same was found when the large-sized brachycephalic breeds were excluded. We found two key confounders for this variable: training experience and the height residual. Both factors were positively associated with trainability, while brachycephalic dogs scored lower on both factors, leading to lower trainability assessment.

In *dog sociability*, where initially no significant difference between head shapes was found (*p* = 0.479), the effect became significant after controlling for the confounders (*p* = 0.049), with brachycephalic dogs being less sociable towards other dogs than dolichocephalic dogs. The same was found when the large-sized brachycephalic breeds were excluded, although on that sample, the global effect of head shape was only at trend level (*p* = 0.092). In this variable, the single key confounder was the dog’s age. This factor was negatively associated with dog sociability, and brachycephalic dogs were younger in our sample, masking their lower score on this trait.

For *calmness* and *boldness*, no marked difference was found between the baseline and controlled models, neither on the full sample nor when the large-sized brachycephalic breeds were excluded.

Regarding behaviour problems, both *jumping up* on people and *pulling the leash*, which were initially not significantly different between head shapes (*p* > 0.3 for both), became significant after controlling for the confounders (*p* < 0.005 for both). In both cases, brachycephalic dogs were less likely to exhibit these behaviour problems compared to both mesocephalic and dolichocephalic dogs. We found largely the same when the large-sized brachycephalic breeds were excluded, although on that sample, the global effect of head shape on jumping up was only at trend level (*p* = 0.096). We found no key confounders for these problems. However, in both cases, the strongest effect was found in weight. In the case of jumping up, only the combined effects of weight and training resulted in the above-mentioned change in significance. Both factors were negatively associated with this problem, and since brachycephalic dogs were lighter and less trained, these factors led to an inflated prevalence of this problem in their case. In pulling the leash, although the combination of weight and training experience had the strongest effect, all pairwise combinations led to significant head shape effects. This probably explains how, when the large-sized brachycephalic breeds were excluded, and so two of the previous confounders, weight and owner experience, were not in the model, the combined effects of the other factors still resulted in the change in the head shape-behaviour association.

The *too reactive when guests arrive* problem was only partially affected by the confounders. The previously significant pairwise difference between brachycephalic and mesocephalic dogs (*p* = 0.012) became nonsignificant (*p* = 0.150) after controlling for the confounders. When the large-sized brachycephalic breeds were excluded, the confounding effect was not partial but full, affecting the global head shape effect, which became nonsignificant (*p* = 0.311), and both previously significant pairwise differences between brachycephalic dogs and the other two groups also vanished (*p* > 0.6 for both). Two confounders were identified for this effect: weight and allowing the dog in bed. Weight was negatively, and allowing the dog into the bed was positively associated with this behavioural problem. As brachycephalic dogs were lighter and more likely to be allowed into the bed of the owner, these factors increased the likelihood of them being seen as overreactive.

Finally, for *not coming back when called*, the only difference we found was that the previously significant pairwise difference between brachycephalic and dolichocephalic dogs (*p* = 0.001) became a trend (0.053) in the controlled model, however it was not an effect strong enough to be tested. When the large-sized brachycephalic breeds were excluded, this pairwise difference became nonsignificant (*p* = 0.203), suggesting a partial mediation effect.

### 3.5. Tests of Mediation

We directly tested the significance of the above-detailed mediation or suppression effects using the lavaan package [44]. In the case of multiple confounders, we run one model for each factor separately and one for their combined effect. The parameter estimates for all models can be found in Table 4. For all except one mediation models, the confidence intervals of the indirect (mediation) effect do not include zero, which is consistent with a statistically significant mediation or suppression effect. The exception was the combined model for *too reactive when guests arrive*. In this model, the two confounders’ effects weakened each other (compared to their individual models), and weight’s indirect effect was no longer significant, suggesting their effects are not independent.

**Figure 2:**
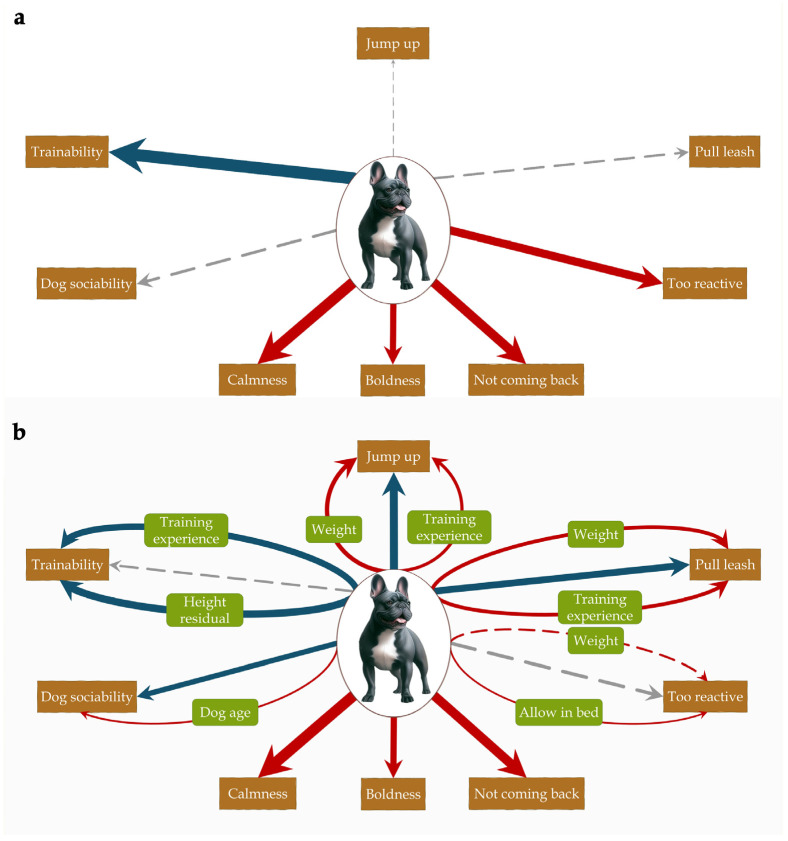
Associations between head shape and behaviour before and after including confounders. (**a**) Baseline associations showing the unmediated total effect of head shape on the behaviours (path c); (**b**) mediation effects (path ab) and adjusted direct effect (path c’) of head shape on the behaviours. Arrows indicate regressions. The thickness of the arrow represents the strength of the association based on standardized regression coefficients; solid arrows indicate significant (*p* < 0.05); dashed arrows non-significant association. The colour of the arrow represents the direction of the association: red: higher score in brachycephalic dogs, grey: no association, blue: lower score in brachycephalic dogs.

To facilitate interpretation of the models, standardized regression coefficients are displayed in Figure 2.

## 4. Discussion

This study aimed to determine whether behavioural differences attributed to head shape, such as shyness [23] and training difficulties [22], are genuinely due to head shape itself or rather the result of interplay with confounding factors. Our hypothesis proposed that body size, demographics, and dog-keeping characteristics mediate the relationship between head shape and behaviour. Most of our findings supported this hypothesis, as we observed that in five behavioural variables, namely trainability, dog sociability, jumping up, pulling the leash, and too reactive when guests arrive, the effect of head shape changed when these mediating factors were included in the model, while the effect remained the same in the case of calmness, boldness, and not coming back when called. To establish this result, we examined three criteria, each of which was analyzed individually.

### 4.1. Baseline Behavioural Differences Between Head Shape Groups

The first objective of this study was to explore the direct associations between head shape and behavioural traits. We found that, according to owners’ reports, brachycephalic dogs were less trainable than both mesocephalic and dolichocephalic dogs. Similarly, both brachycephalic and mesocephalic dogs were less likely to return when called compared to dolichocephalic dogs. These results contrast with prior studies; for instance, Helton [22] found that mesocephalic breeds were perceived as the most trainable, with both extremes of the cephalic index showing lower trainability. However, our findings align with owners’ subjective evaluations of brachycephalic dogs, which often include descriptions of training difficulties and stubbornness [5]. Beyond training-related traits, brachycephalic dogs were also reported to be more reactive to arriving guests compared to both other groups. However, we found no significant differences between head shape groups in jumping up on people or pulling on the leash. Finally, dolichocephalic dogs were rated as the least calm and least bold among all groups. These latter findings align with previous studies, which also reported that a longer head shape is associated with greater fearfulness and increased startle responses [18,19]. In contrast, prior studies have found higher dog-directed aggression in brachycephalic dogs [18,19], which we did not observe in our sample.

Overall, our findings did not provide strong support for the assumption that brachycephalic dogs display more desirable behavioural traits for companionship [5], which could potentially compensate for their physical and welfare challenges. However, it is worth noting that some of the traits that are typically considered positive for companionship (e.g., sociability, interactivity, visual communication with humans, playfulness, or affectionate behaviour) were not explicitly captured by the behavioural variables measured in this study.

### 4.2. Associations Between Potential Confounding Factors and Head Shape

Next, we examined the relationships between head shape, body size, demographics, and dog-keeping characteristics as potential confounders. Our findings support and extend the previous literature, demonstrating that brachycephalic dogs are indeed kept differently from dogs with longer head shapes.

As expected, brachycephalic dogs were more likely to be kept solely as family companions and less likely to have practical roles, and their environment and keeping practices reflect this status. They were also smaller in body size than the other two head shape groups. Additionally, their average age was lower, likely reflecting their shorter life expectancy [3], which may have led to older dogs being underrepresented in the sample [5].

While the majority of categorical factors showed significant differences between head shape groups, the magnitude of most differences was relatively small. However, seven factors exhibited substantial (>10%) differences, with the most pronounced being training experience: nearly half of brachycephalic dogs had not received any formal training, compared to less than 30% in the other two groups. Additionally, brachycephalic dogs were more likely to be allowed on their owners’ beds, their owners spent more time with them, they were more often kept exclusively indoors (in line with [13]), and they were walked less frequently than the other two groups. These patterns are consistent with owners’ expectations of these breeds as suited to a sedentary lifestyle with limited space [5]. However, reduced walking time could also be a direct consequence of their health problems rather than solely an owner-driven decision [49].

Owners of brachycephalic dogs also differed demographically from those of other head shape groups; they were more likely to be women, younger, less educated, living alone, and first-time dog owners. These findings are consistent with prior studies [6,13], and may be partly explained by psychological mechanisms such as increased sensitivity to infantile features (i.e., the baby schema effect; [50]). Previous work has also found that people with a positive attitude towards brachycephalic dogs tend to score higher in agreeableness, conscientiousness, and dog-directed empathy [6], though this does not necessarily reflect the personality profile of actual owners of brachycephalic dogs, which remains to be unmeasured. Nevertheless, it is possible that individuals with certain personality profiles are drawn to brachycephalic breeds not only because of their appearance but also because they perceive the behavioural tendencies associated with such breeds, such as sociability, family-friendliness, and emotional dependence, as congruent with their own personality profile. Future studies should explore whether a potential personality similarity between dog and owner contributes to breed preference and emotional attachment.

In summary, brachycephalic dogs are indeed kept and treated as child-like companion animals, either because owners select them for this purpose or because their infant-like appearance and/or behaviour elicit such treatment. These differences in keeping characteristics may, in turn, influence the behaviour of these dogs.

### 4.3. Associations Between Potential Confounding Factors and Behaviour

Our results confirm that, as found in numerous previous studies, environmental, keeping, and demographic factors are associated with the examined behavioural traits. Regarding personality factors, prior research has already explored these relationships extensively [29,42], and our findings largely align with those studies.

Briefly, both age and training experience had significant effects on personality traits and behavioural problems as well, but in different ways. Age had a negative impact on personality traits (older dogs showed less favourable personality traits) but a positive effect on behavioural problems (fewer issues with increasing age). In contrast, training experience had a uniformly positive effect on both: higher training was associated with more favourable personality traits and fewer behavioural problems. These findings align with prior research [24,27,34]. Additionally, neutering negatively impacted personality traits, while time spent with the owner and younger adoption age were associated with more favourable personality traits, also consistent with the literature [32]. However, we cannot rule out the possibility that older adoption age reflects pre-existing behavioural problems that led to relinquishment, rather than being a causal factor in personality development.

Regarding behavioural problems, beyond training and age, owner experience and body weight also played significant roles: more experienced owners reported fewer behavioural issues, and lighter dogs exhibited more behavioural problems. Interestingly, although height and weight were highly correlated, they were associated with different behavioural traits. Height residual (controlling for weight) was significantly related only to trainability: gracile dogs, i.e., those that are taller than what is typical for their weight, such as greyhounds, were more trainable than stocky built ones. This is consistent with prior research claiming a positive association between height and trainability [18,39]. In contrast, behavioural problems were primarily associated with weight, supporting previous findings that lighter dogs exhibit more undesirable behaviours, including aggression, hyperactivity, attention-seeking, and disobedience [18,19,26,32,37].

### 4.4. Tests of Mediation

Since several factors met both criteria of being a potential mediator or suppressor (i.e., were significantly associated with both head shape and behaviour), we were able to directly test our hypothesis regarding the extent to which these confounders act as mediators or suppressors between head shape and behaviour. We found such confounding effects in two personality traits (trainability and dog sociability) and three behavioural problems (jumping up, pulling the leash, too reactive). In four of these five cases, controlling for the confounding factors resulted in a positive shift in behaviour; the exception was dog sociability.

Mediation effects offer a mechanism by which the predictor (head shape) influences the outcome (behaviour). Such an effect was observed in two behavioural traits. Trainability was initially lower in brachycephalic dogs compared to the other two groups, but this difference was fully mediated by two factors: height residual and training experience. These two factors were positively associated with trainability, and brachycephalic dogs had lower values for both, suggesting that their reduced trainability is due to these factors rather than head shape itself. When both factors were included in the model, their effects strengthened each other. Although previous studies showed that smaller dogs receive less formal training [28,37], suggesting that the indirect effect of height on trainability should be at least partly mediated by the training experience itself, our results show instead that the two factors work through different mechanisms.

For too reactive when guests arrive, we observed a partial mediation effect. Brachycephalic dogs were reported to be more reactive than both other groups, but their difference from mesocephalic dogs was mediated by two factors: weight and being allowed on the owner’s bed. Owners of lighter dogs and those who allowed their dogs to sleep in their beds reported more problems, and since brachycephalic dogs were both lighter and more often allowed in bed, these factors contributed to their increased reactivity. Here, when both factors (weight and being allowed on the owner’s bed) were included in the model, their effects weakened each other, and weight’s indirect effect became nonsignificant. This indicates that lower weight increases reactivity, at least partly, due to owners’ spoiling behaviours.

Conversely to mediation, suppression effect occurs when a third variable obscures the direct effect of the predictor (head shape) on the outcome (behaviour). Controlling for the suppressor removes irrelevant variance from the predictor, thereby revealing the true relationship between the predictor and the outcome that was not apparent before. We found three suppression effects. For jumping up and pulling the leash, weight and training experience jointly masked head shape differences. Both factors were negatively associated with these behavioural problems, and because brachycephalic dogs were lighter and less trained, these factors obscured the fact that short-headed dogs were actually less prone to these behaviours. The similarity in suppression effects across these problems also suggests a shared underlying motivation.

In dog sociability, the initial absence of head shape differences was primarily due to age differences across the head shape groups. Dog age was negatively associated with sociability towards other dogs, and brachycephalic dogs were younger, which masked their generally lower sociability. This finding aligns with prior studies reporting higher dog-directed aggression in shorter-headed dogs [5,18]. This behaviour is somewhat unique, as it is the only one that changed in a negative direction after controlling for confounding factors. Moreover, it was also the only case where body size did not appear to act as a confounding factor, even though it would be a logical assumption, considering that smaller dogs are disproportionately more aggressive [26,30]. This raises the question of why brachycephalic dogs tend to be more aggressive towards other dogs, independent of body size. One possibility is communication difficulties: just as humans struggle to interpret their facial expressions [51], other dogs may also find their facial signals harder to read. Another possibility is genetic predisposition, as several brachycephalic breeds were initially bred for dog fighting [22] and other tasks requiring higher aggression (e.g., bull-type dogs). A third, not mutually exclusive explanation is that brachycephalic dogs may experience chronic discomfort due to health issues associated with their conformation, and such persistent pain has been suggested to underlie a range of behavioural problems, including aggression [52]. However, further research is needed to explore this issue.

Finally, in the remaining three behavioural variables examined (calmness, boldness, and obedience when called), neither body size, nor keeping practices, nor environmental factors mediated or suppressed the differences between head shape groups. The lower calmness and boldness, as well as higher obedience when called in dolichocephalic dogs, seem unrelated to body size, environment, or keeping practices—at least the factors we measured. These traits may be direct effects of genetics and skull morphology (as has been suggested for fearfulness [18]), further supporting that selective breeding for morphological and behavioural traits are also linked to brain anatomy and function [53]. Alternatively, they could be influenced by unmeasured factors such as socialization or traumatic experiences, which might also correlate with head shape.

### 4.5. Limitations

Unlike many past studies that focused on only a few breeds and measured only one or a few behavioural traits, our research benefits from a large sample size, multiple breeds (while controlling for breed popularity), and a wide range of behavioural measures. However, our study has certain limitations. First, the behavioural data were obtained through owner reports, which are inherently subjective. This subjectivity may disproportionately bias assessments of dogs with different head shapes, as previous studies have shown that “cute” dogs tend to be perceived as having more desirable personality traits [9,11]. This bias is particularly concerning when evaluating behavioural problems, where it was not sufficient for a behaviour to be theoretically problematic (i.e., undesirable); the owners also needed to perceive it as unpleasant, annoying, or difficult to manage [33]. Consequently, owners of “cute” brachycephalic dogs may have underreported some behavioural issues simply because they did not recognize them as problematic. Moreover, since owners of brachycephalic dogs in our sample also tended to have less dog-keeping experience, we cannot exclude the possibility that their lower ability to read canine body language [54] might have contributed to the differences we found among the breed groups. Additionally, aside from dog-related experience, the psychological characteristics of the owners may also systematically influence breed choice and behaviour reporting. Although we did not measure these characteristics directly, emotionally motivated preferences, such as a heightened desire to care for vulnerable animals, for instance, might contribute to both the selection of brachycephalic dogs and biased perception of their behaviour, a possibility that future research should investigate more directly. Finally, as our data derive from a convenience sample of volunteer owners, it is possible that owners who are more satisfied with their dogs or who experience fewer health or behavioural problems were more likely to participate. This selection bias may disproportionately affect brachycephalic dogs, as owners of dogs with serious health or behavioural issues might be underrepresented, potentially limiting the generalizability of our findings.

Aside from potential response biases, using discrete head shape groups instead of a continuous cephalic index scale is probably the most obvious limitation of this study. Classifying head shapes into groups based on arbitrary cut-off values has been criticized as overly simplistic [55]. However, this approach is still justifiable when having only breed-level data. As the cephalic index itself varies even within breeds, using a continuous scale would not necessarily eliminate classification challenges. Moreover, we defined our head shape categories using non-continuous thresholds, ensuring that the groups were meaningfully distinct rather than overlapping at arbitrary thresholds. While we acknowledge that a continuous approach and individual measurements may be preferable in most contexts, our robust categorical classification allowed us to capture large-scale behavioural trends and provide practical and easily interpretable results, while minimizing the risk of artificial gradations within a highly variable trait.

## 5. Conclusions

In summary, the mediation and suppression patterns of the examined behavioural traits suggest four key conclusions. First, not everything is the effect of head shape that looks like it, and vice versa. In two cases, apparent behavioural differences are actually due to confounding factors such as body size and training experience. In three others, real effects of head shape remain hidden until these confounders are accounted for. Overall, head shape directly influences the behaviour in six out of eight variables, but only in the cases where dolichocephalic dogs differ from the other groups are these direct effects obvious in everyday life. Second, brachycephalic dogs may inherently exhibit behaviours that appeal to owners, e.g., not jump up or pull the leash, but these positive behaviours are often not evident in everyday life due to the effect of confounders. Third, among all dog-keeping factors, only training experience plays a crucial role as a confounding factor in the head shape-behaviour associations. This indicates that, even though short-headed dogs are kept differently (for instance, more often kept indoors) than other head shapes, their predisposition for positive behaviours is primarily genetic or at least morphology-related and not the result of their lapdog-like keeping characteristics. Finally, there seems to be conflicting selection pressures between small size and brachycephalic head shape. Selection for both traits—short skull and small body—has been suggested to alter how dogs perceive and process stimuli [16,18], and to increase neotenous behavioural characteristics [17,18]. However, while in the case of small body size, this selection seems to lead to more undesirable behaviours, the selection for brachycephaly appears to favour behaviours that are more positive to owners. Consequently, in small-sized brachycephalic dogs, these two selection pressures may result in a complex behavioural trade-off, where traits associated with small size (e.g., increased excitability, fearfulness, and attention-seeking) coexist with traits favoured by brachycephaly (e.g., sociability, interactivity, calmness, and dependence). This interplay may shape their unique behavioural profile, which differs from both small non-brachycephalic dogs and large brachycephalic breeds. However, our findings also raise a provocative question for future research: could the calm, dependent temperament of brachycephalic breeds be less a result of deliberate selection and more a by-product of chronic health problems that limit their activity and reactivity? If so, their popularity might be driven, at least in part, by a perceived helplessness that elicits caregiving responses from humans [6,14].

It is also worth noting that our findings—including behavioural and keeping differences between head shapes, and mediation or suppression effects—remained largely unchanged even after excluding large-bodied brachycephalic dogs, which comprised nearly one-third of the brachycephalic sample. This suggests that the observed differences are primarily driven by smaller brachycephalic dogs. However, it is important to note that most of our models already controlled for the effect of size, meaning that removing large brachycephalic dogs did not introduce new variation but rather reinforced what was already evident from the statistical controls. Nevertheless, these results highlight the robustness of our findings, indicating that they are not merely an artefact of size variation among brachycephalic dogs but genuinely reflect the unique behavioural and keeping characteristics of small brachycephalic dogs. However, our findings also urge caution, as the observed associations between head shape, behaviour, and keeping practices should not be generalized to all brachycephalic dogs, only to small-sized ones.

Understanding the interplay between head shape, body size, behaviour, and keeping practices is crucial from multiple perspectives. From a scientific perspective, our findings contribute to a more nuanced understanding of how morphology shapes dog behaviour, emphasizing the need to consider head shape and body size together rather than treating brachycephalic dogs as a homogeneous group. For current and prospective owners, our research provides valuable insights into the behavioural tendencies and care needs of brachycephalic breeds, helping them make informed decisions. For example, our results highlight that even for small-sized brachycephalic dogs, proper training—rather than indulgence—is essential for fostering positive behaviours. Given the rising popularity of these breeds despite their well-documented health and welfare challenges, a better understanding of their behaviour could support responsible ownership and training practices, ultimately contributing to the long-term welfare of these dogs. Finally, our findings may also be informative for breeders and welfare agencies seeking to improve the well-being of brachycephalic dogs by raising awareness of the trade-offs between head shape and small size.

## Figures and Tables

**Table 1:** Baseline behavioural differences across head shape groups. Generalized linear models (GLMs) were conducted using linear or binomial logistic distributions, depending on the behavioural trait, with head shape as the sole fixed factor. Parameter estimates for the pairwise comparisons are also presented. Significant differences are marked in italics.

Global Head Shape Effect			Pairwise Comparisons					
Behaviour	Wald χ^2^	*p* Value	Dog Group	B	SE	Wald χ^2^	*p* Value	Exp(B) (95% Conf Int)
*Calmness*	*18.139*	*<0.001*						
			*Brachy > Dolicho*	*−0.200*	*0.0480*	*17.701*	*<0.001*	*0.819 (0.746–0.899)*
			Brachy vs. Meso	−0.060	0.0410	2.153	0.142	0.942 (0.869–1.020)
			*Dolicho < Meso*	*0.140*	*0.0450*	*9.563*	*0.002*	*1.150 (1.053–1.256)*
*Trainability*	*26.194*	*<0.001*						
			*Brachy < Dolicho*	*0.212*	*0.0460*	*21.259*	*<0.001*	*1.236 (1.130–1.353)*
			*Brachy < Meso*	*0.181*	*0.0420*	*18.420*	*<0.001*	*1.199 (1.103–1.302)*
			Dolicho vs. Meso	−0.031	0.0430	0.502	0.479	0.970 (0.891–1.056)
Dog sociability	1.470	0.479						
			Brachy vs. Dolicho	0.046	0.0460	0.960	0.327	1.047 (0.955–1.146)
			Brachy vs. Meso	0.047	0.0420	1.260	0.262	1.048 (0.965–1.138)
			Dolicho vs. Meso	0.002	0.0430	0.001	0.971	1.002 (0.920–1.090)
*Boldness*	*9.489*	*0.009*						
			*Brachy > Dolicho*	*−0.119*	*0.0470*	*6.336*	*0.012*	*0.888 (0.809–0.974)*
			Brachy vs. Meso	0.012	0.0410	0.081	0.776	1.012 (0.933–1.097)
			*Dolicho < Meso*	*0.131*	*0.0450*	*8.583*	*0.003*	*1.140 (1.044–1.244)*
Jumping up	0.214	0.898						
			Brachy vs. Dolicho	−0.046	0.1160	0.158	0.691	0.955 (0.761–1.198)
			Brachy vs. Meso	−0.001	0.1010	0.000	0.996	0.999 (0.821–1.217)
			Dolicho vs. Meso	0.046	0.1070	0.182	0.669	1.047 (0.849–1.290)
Pulling the leash	2.125	0.346						
			Brachy vs. Dolicho	0.117	0.1150	1.034	0.309	1.124 (0.897–1.409)
			Brachy vs. Meso	−0.035	0.1020	0.121	0.728	0.965 (0.790–1.179)
			Dolicho vs. Meso	−0.153	0.1060	2.074	0.150	0.858 (0.697–1.057)
*Too reactive*	*23.539*	*<0.001*						
			*Brachy > Dolicho*	*−0.652*	*0.1350*	*23.240*	*<0.001*	*0.521 (0.399–0.679)*
			*Brachy* vs. *Meso*	*−0.306*	*0.1250*	*5.990*	*0.014*	*0.736 (0.576–0.941)*
			*Dolicho < Meso*	*0.346*	*0.1170*	*8.749*	*0.003*	*1.414 (1.124–1.778)*
*Not coming back*	*11.975*	*0.003*						
			*Brachy > Dolicho*	*−0.354*	*0.1090*	*10.564*	*0.001*	*0.702 (0.567–0.869)*
			Brachy vs. Meso	−0.068	0.0960	0.510	0.475	0.934 (0.775–1.126)
			*Dolicho < Meso*	*0.286*	*0.1000*	*8.194*	*0.004*	*1.331 (1.094–1.618)*

**Table 2:** Comparison of demographic and dog-keeping factors between head shape groups. For each categorical variable, the proportion of dogs in each category is presented separately for each head shape group. Significant group differences, as indicated by the Chi-squared tests with z post hoc tests, are highlighted by marking the category with the larger proportion in each group in bold. Only differences with a *p*-value < 0.00037 are considered significant and are marked in italics. For pairwise significant differences, the absolute magnitude of the difference is provided as a measure of effect size. B: brachycephalic; M: mesocephalic; D: dolichocephalic.

Factors in Both Surveys	Categories	B (N = 1786)	M (N = 2388)	D (N = 1439)	Statistics	Absolute Magnitude of the Differences
Dogs’ age in years (mean ± SD)	–	2.94 (±2.87)	3.23 (±2.91)	3.68 (±3.18)	*H = 59.880*	B vs. M: 0.30 years; B vs. D: 0.74 years; M vs. D: 0.45 years
					*p < 0.001*	
Dogs’ weight in kg (mean ± SD)	–	14.99 (±14.32)	24.72 (±15.70)	27.63 (±9.29)	*H = 975.790*	B vs. M: 16.21 kg; B vs. D: 22.91 kg; M vs. D: 6.70 kg
					*p < 0.001*	
Dogs’ height in cm (mean ± SD) *	–	34.91 (±14.18)	51.12 (±13.63)	57.82 (±10.66)	*H = 1954.813*	B vs. M: 9.73 cm; B vs. D: 12.64 cm; M vs. D: 2.91 cm
					*p < 0.001*	
Dogs’ sex	male	56.1%	59.5%	56.8%	χ^2^ = 5.425	
	female	43.9%	40.5%	43.2%	*p* = 0.066	
Dogs’ neuter status	intact	**74.5%**	66.5%	69.5%	*χ* ^2^ * = 31.632*	B vs. M: 8.07%; B vs. D: 5.03%
	neutered	25.5%	**33.5%**	**30.5%**	*p < 0.001*	
Dogs’ age at acquisition	bred by owner	1.4%	1.8%	2.5%	*χ* ^2^ * = 50.378*	
	2–12 weeks	62.5%	**66.7%**	58.6%	*p < 0.001*	B vs. M: 4.18%; M vs. D: 8.08%
	3–12 months	**24.2%**	18.8%	21.3%		B vs. M: 5.39%
	>1 year	11.9%	12.7%	**17.6%**		B vs. D: 5.66%; M vs. D: 4.85%
Dogs’ training experience	no training	**48.4%**	28.4%	28.8%	*χ* ^2^ * = 302.570*	B vs. M: 20.00%; B vs. D: 19.66%
	1 activity	25.4%	23.6%	**27.7%**	*p < 0.001*	M vs. D: 4.04%
	2 activities	17.4%	**24.2%**	**21.7%**		B vs. M: 6.75%; B vs. D: 4.27%
	3 or more activities	8.7%	**23.8%**	**21.9%**		B vs. M: 15.05%; B vs. D: 13.16%
Owners’ sex	man	17.9%	19.3%	**23.3%**	*χ* ^2^ * = 16.025*	B vs. D: 5.49%; M vs. D: 4.09%
	woman	**82.1%**	**80.7%**	76.7%	*p < 0.001*	
Owners’ age	≤18 years	5.0%	5.1%	3.5%	*χ* ^2^ * = 93.972*	
	19–30 years	**37.2%**	27.1%	23.8%	*p < 0.001*	B vs. M: 10.13%; B vs. D: 13.34%
	31–60 years	54.9%	**65.4%**	**69.4%**		B vs. M: 10.44%; B vs. D: 14.43%; M vs. D: 3.99%
	>60 years	2.9%	2.5%	3.3%		
Owners’ education	primary school	23.0%	21.3%	20.7%	*χ* ^2^ * = 32.147*	
	secondary school	38.7%	42.3%	39.7%	*p < 0.001*	
	college	**28.8%**	24.6%	24.9%		B vs. M: 4.21%; B vs. D: 3.96%
	university	9.5%	11.7%	**14.7%**		B vs. D: 5.20%; M vs. D: 2.94%
N of previous dogs	no previous dog	**45.1%**	**44.8%**	33.6%	*χ* ^2^ * = 62.779*	B vs. D: 11.50%; M vs. D: 11.18%
	1 dog	24.5%	25.7%	27.5%	*p < 0.001*	
	2 dogs	14.4%	14.4%	**18.5%**		B vs. D: 4.14%; M vs. D: 4.09%
	3 or more dogs	16.0%	15.1%	**20.4%**		B vs. D: 4.34%; M vs. D: 5.26%
Purpose of keeping the dog	family member only	**56.2%**	**43.8%**	38.8%	*χ* ^2^ * = 109.992*	B vs. M: 12.31%; B vs. D: 17.38%; M vs. D: 5.07%
	family member + other	38.4%	**48.7%**	**52.3%**	*p < 0.001*	B vs. M: 10.33%; B vs. D: 13.92%
	not family member	5.4%	7.4%	**8.9%**		B vs. D: 3.46%
N of people in the household	only 1 person	**12.7%**	9.6%	10.8%	*χ* ^2^ * = 22.914*	B vs. M: 3.08%
	2 people	46.0%	42.6%	45.9%	*p < 0.001*	
	3 or more people	41.3%	**47.8%**	43.3%		B vs. M: 6.52%; M vs. D: 4.52%
N of dogs in the household	no other dog	66.7%	68.3%	63.4%	χ^2^ = 11.630	
	1 other dog	20.9%	19.4%	23.8%	*p* = 0.020	
	≥2 other dogs	12.4%	12.3%	12.7%		
**Factors only in Survey 1**	**Categories**	**B (N = 1064)**	**M (N = 1281)**	**D (N = 833)**	**Statistics**	**Absolute magnitude of the differences**
Hours spent with the dog/day	≤3 h	18.0%	**27.3%**	**27.4%**	*χ* ^2^ * = 33.319*	B vs. M: 9.28%; B vs. D: 9.33%
	>3 h	**82.0%**	72.7%	72.6%	*p < 0.001*	
Frequency of playing/week	1–5 days	15.4%	21.5%	19.0%	χ^2^ = 14.324	
	6–7 days	84.6%	78.5%	81.0%	*p* = 0.0007	
**Factors only in Survey 2**	**Categories**	**B (N = 745)**	**M (N = 1124)**	**D (N = 630)**	**Statistics**	**Absolute magnitude of the differences**
N of children in the household	1 or more	79.3%	76.0%	82.0%	χ^2^ = 8.953	
	none	20.7%	24.0%	18.0%	*p* = 0.011	
Where the dog is kept	only indoors	**81.1%**	68.3%	71.9%	*χ* ^2^ * = 37.968*	B vs. M: 12.75%; B vs. D: 9.17%
	in- and outdoors	17.4%	**28.8%**	**25.2%**	*p < 0.001*	B vs. M: 11.38%; B vs. D: 7.79%
	only outdoors	1.5%	2.8%	2.9%		
Hours spent walking the dog	<1 h	**21.5%**	10.1%	9.2%	*χ* ^2^ * = 71.018*	B vs. M: 11.33%; B vs. D: 12.27%
	1–3 h	71.3%	**77.2%**	**81.0%**	*p < 0.001*	B vs. M: 5.95%; B vs. D: 9.68%
	>3 h	7.2%	**12.6%**	9.8%		B vs. M: 5.39%
Hours spent playing/day	≤1 h	66.7%	67.8%	67.8%	χ^2^ = 0.276	
	>1 h	33.3%	32.2%	32.2%	*p* = 0.871	
Buy gifts for the dog	yes	27.1%	29.7%	34.8%	χ^2^ = 9.708	
	no	72.9%	70.3%	65.2%	*p* = 0.008	
Allow the dog into the bed	yes	46.4%	**66.2%**	**63.7%**	*χ* ^2^ * = 78.176*	B vs. M: 19.75%; B vs. D: 17.21%
	no	**53.6%**	33.8%	36.3%	*p < 0.001*	

* For reference only, in the analyses, the height residual was examined instead of raw height.

**Table 3:** Behaviour differences between head shape groups after controlling for all potential confounding factors. Generalized linear models were run using linear or binomial logistic distribution (depending on the behavioural trait), with head shape and all potential confounders significantly associated with both head shape and behaviour as fixed factors. Parameter estimates of the pairwise comparisons are also provided. Differences where the significance status changed compared to the baseline model are marked in italics.

Global Head Shape Effect			Pairwise Comparisons					
Behaviour	Wald χ^2^	*p* Value	Dog Group	B	SE	Wald χ^2^	*p* Value	Exp(B) (95% Conf Int)
Calmness	14.814	0.001						
			Brachy > Dolicho	−0.174	0.047	13.489	<0.001	0.841 (0.766–0.922)
			Brachy vs. Meso	−0.032	0.041	0.609	0.435	0.969 (0.894–1.049)
			Dolicho < Meso	0.142	0.045	9.920	0.002	1.152 (1.055–1.258)
*Trainability*	*0.757*	*0.685*						
			*Brachy* vs. *Dolicho*	*0.048*	*0.056*	*0.734*	*0.391*	*1.049 (0.940–1.170)*
			*Brachy* vs. *Meso*	*0.032*	*0.047*	*0.464*	*0.496*	*1.032 (0.942–1.131)*
			*Dolicho* vs. *Meso*	*−0.016*	*0.043*	*0.141*	*0.707*	*0.984 (0.905–1.070)*
*Dog sociability*	*6.052*	*0.049*						
			*Brachy < Dolicho*	*0.110*	*0.045*	*5.955*	*0.015*	*1.116 (1.022–1.219)*
			Brachy vs. Meso	0.066	0.041	2.525	0.112	1.068 (0.985–1.158)
			Dolicho vs. Meso	−0.044	0.041	1.147	0.284	0.957 (0.882–1.037)
Boldness	6.750	0.034						
			Brachy vs. Dolicho	−0.087	0.047	3.471	0.062	0.917 (0.836–1.005)
			Brachy vs. Meso	0.027	0.041	0.42	0.517	1.027 (0.948–1.113)
			Dolicho < Meso	0.114	0.044	6.582	0.010	1.120 (1.027–1.222)
*Jumping up*	*10.803*	*0.005*						
			*Brachy < Dolicho*	*0.363*	*0.128*	*8.035*	*0.005*	*1.437 (1.118–1.847)*
			*Brachy < Meso*	*0.329*	*0.11*	*8.946*	*0.003*	*1.390 (1.120–1.725)*
			Dolicho vs. Meso	−0.034	0.111	0.092	0.762	0.967 (0.778–1.202)
*Pulling the leash*	*16.222*	*<0.001*						
			*Brachy < Dolicho*	*0.518*	*0.129*	*16.222*	*<0.001*	*1.678 (1.304–2.159)*
			*Brachy < Meso*	*0.264*	*0.112*	*5.535*	*0.019*	*1.302 (1.045–1.622)*
			*Dolicho > Meso*	*−0.254*	*0.111*	*5.254*	*0.022*	*0.776 (0.624–0.964)*
Too reactive	12.983	0.002						
			Brachy > Dolicho	−0.497	0.143	12.029	0.001	0.608 (0.459–0.806)
			*Brachy* vs. *Meso*	*−0.190*	*0.132*	*2.075*	*0.150*	*0.827 (0.639–1.071)*
			Dolicho < Meso	0.308	0.118	6.768	0.009	1.360 (1.079–1.715)
Not coming back	5.484	0.064						
			Brachy vs. Dolicho	−0.220	0.113	3.756	0.053	0.803 (0.643–1.002)
			Brachy vs. Meso	0.004	0.100	0.002	0.964	1.004 (0.826–1.222)
			Dolicho < Meso	0.224	0.102	4.793	0.029	1.251 (1.024–1.529)

**Table 4:** Unstandardized parameter estimates (regression coefficients and standard errors [se] for path a–c’), and 95% confidence interval) for the mediation models for the head shape-behaviour association. Path a is the relationship between head shape and the proposed mediator. Path b is the relationship between the proposed mediator and behaviour. All regression coefficients of these two paths are significant. Path c is the unmediated direct effect of head shape on the behaviour (also called total effect). Path c’ is the direct effect of head shape when the indirect (mediation) effect is included in the model. The mediation effect is calculated as path a × path b. * *p* < 0.05; ** *p* < 0.01; *** *p* < 0.001.

Behaviour	Mediator	Path a (se)	Path b (se)	Path c (se)	Path c’ (se)	Mediation Effect (95% CI)
Trainability	Training experience	0.280 (0.025)	0.085 (0.006)	0.046 (0.010) ***	0.022 (0.009) *	0.024 (0.019–0.030) ***
	Height residual	0.710 (0.016)	0.046 (0.011)	0.046 (0.010) ***	0.013 (0.012)	0.033 (0.017–0.048) ***
	Combined	Training experience: 0.464 (0.050)	0.082 (0.008)	0.046 (0.010) ***	−0.014 (0.021)	0.038 (0.028–0.050) ***
		Height residual: 0.879 (0.029)	0.050 (0.014)			0.044 (0.019–0.066) ***
Dog sociability	Dog age	0.305 (0.121)	−0.045 (0.004)	0.024 (0.022)	0.038 (0.020)	−0.014 (−0.024–−0.002) *
Jumping up	Weight + Training experience	Weight: 8.918 (0.691)	−0.003 (0.001)	−0.005 (0.013)	0.052 (0.022) *	−0.029 (−0.042–−0.017) ***
		Training experience: 0.498 (0.052)	−0.043 (0.010)			−0.022 (−0.034–−0.012) ***
Pulling the leash	Weight + Training level	Weight: 8.918 (0.698)	−0.003 (0.001)	0.012 (0.013)	0.045 (0.023) *	−0.025 (−0.036–−0.013) ***
		Training experience: 0.498 (0.052)	−0.049 (0.009)			−0.025 (−0.036–−0.014) ***
Too reactive when guests arrive	Weight	8.918 (0.693)	−0.002 (0.001)	−0.045 (0.017) **	−0.029 (0.018)	−0.016 (−0.028–−0.005) **
	Allow the dog into bed	−0.197 (0.023)	0.079 (0.017)	−0.045 (0.017) **	−0.029 (0.018)	−0.016 (−0.024–−0.008) ***
	Combined	Weight: 8.918 (0.694)	−0.001 (0.001)	−0.045 (0.017) **	−0.019 (0.019)	−0.012 (−0.024–0.000)
		Allow the dog into bed: −0.134 (0.023)	0.070 (0.018)			−0.009 (−0.015–−0.004) **

## Data Availability

All data required to replicate the analyses presented in this study are publicly available on Figshare (DOI: 10.6084/m9.figshare.28815485) at https://doi.org/10.6084/m9.figshare.28815485.

## Supplementary Materials

The following supporting information can be downloaded at: https://www.mdpi.com/article/10.3390/ani15152221/s1, Table S1. The list of breeds included in the study; Table S2. The four personality traits (from Survey 1) and the four behavioural problems (from Survey 2); Table S3. Baseline behavioural differences across head shape groups when large-sized brachycephalic dogs are excluded from the sample; Table S4. Comparison of demographic and dog-keeping factors between head shape groups when large-sized brachycephalic dogs are excluded from the sample; Table S5. Associations between the potential confounding factors and behaviour; Table S6. Associations between the potential confounding factors and behaviour when large-sized brachycephalic dogs are excluded from the sample.
