# Supplementary material for: Selection for Short-Nose and Small Size Creates a Behavioural Trade-Off in Dogs
Source: Animals (Basel). 2025 Jul 28;15(15):2221. doi: 10.3390/ani15152221 (PMC12345462; doi:10.3390/ani15152221)
Supplement: Supplementary file 1 [file animals-15-02221-s001.zip › animals-3756120-supplementary.pdf]

Supplementary Information
Title: Selection for short-nose and small size creates a behavioural trade-off in dogs
Journal: Animals
Authors: Borbála Turcsán*, Enikő Kubinyi
Primary affiliation of the corresponding author: MTA-ELTE Lendület "Momentum" Companion Animal Research Group, Department of Ethology, ELTE Eötvös Loránd University, Budapest, Hungary
email address of the corresponding author: turcsan.borbala@ttk.elte.hu

Supplementary Table S1. The list of breeds included in the study. Their sample sizes in Survey 1 (S1) and Survey 2 (S2), sex ratio, mean age, mean cephalic index (CI) and head shape categorization. A maximum for the number of individuals / breed had been established to control for the popularity of the breeds (max N = 100 / survey). From the breeds with more representatives than this cut-off point a random sample was selected for the final dataset. N = 64 dogs participated in both surveys. When calculating the total number individuals, these dogs were counted only once.

Breed	N S1	N S2	N Total	Sex ratio (male %)	Age (mean)	CI (mean)	Head shape group
Afghan Hound	3	2	5	40.0%	7.8	49.41	dolichocephalic
Airedale Terrier	31	37	67	61.2%	3.7	49.18	dolichocephalic
Akita Inu	22	6	28	46.4%	3.8	57.32	mesocephalic
Alaskan Malamute	11	8	18	77.8%	4.6	58.08	mesocephalic
American Bulldog	68	15	83	59.0%	2.3	78.34	brachycephalic
American Cocker Spaniel	16	1	17	64.7%	5.7	59.42	mesocephalic
American Pit Bull Terrier	58	21	78	59.0%	4.9	58.61	mesocephalic
American Staffordshire Terrier	102	39	139	53.2%	4.9	67.04	brachycephalic
Australian Cattle Dog (Heeler)	11	10	21	42.9%	1.9	61.62	mesocephalic
Australian Kelpie	3	5	8	62.5%	2.1	56.75	mesocephalic
Australian Shepherd	100	100	200	57.0%	2.5	54.96	mesocephalic
Australian Terrier	5	10	14	42.9%	3.4	57.89	mesocephalic
Basenji	4	3	7	85.7%	5.5	55.83	mesocephalic
Basset Hound	8	5	13	38.5%	4.0	49.77	dolichocephalic
Beagle	100	100	200	57.5%	2.5	59.01	mesocephalic
Belgian Shepherd Dog - Groenendael	9	9	17	47.1%	4.1	44.16	dolichocephalic
Belgian Shepherd Dog - Tervueren	6	6	12	58.3%	4.8	48.41	dolichocephalic
Bernese Mountain Dog (Berner Sennenhund)	100	100	200	55.0%	3.0	60.13	mesocephalic
Bolognese	8	6	13	76.9%	3.4	72.82	brachycephalic
Border Collie	100	100	200	57.3%	3.3	55.68	mesocephalic
Borzoi	5	2	7	71.4%	4.1	38.66	dolichocephalic
Boston Terrier	5	8	13	76.9%	2.2	93.42	brachycephalic
Bouvier des Flandres	8	10	16	56.3%	4.0	46.00	dolichocephalic
Boxer	100	100	200	49.0%	3.0	68.91	brachycephalic
Bull Terrier	20	15	35	57.1%	3.6	55.24	mesocephalic
Bulldog	67	40	107	52.3%	2.3	91.34	brachycephalic
Bullmastiff	8	19	27	59.3%	3.1	79.08	brachycephalic
Cane Corso	11	28	38	60.5%	2.0	60.90	mesocephalic
Cavalier King Charles Spaniel	25	36	60	61.7%	2.5	76.83	brachycephalic
Chihuahua	103	62	162	56.2%	2.5	72.89	brachycephalic
Chinese Crested Dog	8	4	11	90.9%	3.6	59.86	mesocephalic
Collie, Rough	64	44	105	55.2%	4.0	46.43	dolichocephalic
Doberman Pinscher	100	82	182	59.3%	3.1	46.01	dolichocephalic
Dogo Argentino	10	9	19	47.4%	3.8	60.60	mesocephalic
English Cocker Spaniel	102	81	181	58.6%	4.2	48.23	dolichocephalic
English Mastiff	3	13	16	50.0%	3.5	70.73	brachycephalic
English Setter	11	11	22	54.5%	5.1	43.57	dolichocephalic
Finnish lapphund	1	0	1	0.0%	5.0	57.32	mesocephalic
Fox Terrier, Smooth	9	5	14	71.4%	4.5	45.03	dolichocephalic
French Bulldog	81	105	181	65.2%	2.1	100.51	brachycephalic
German Pointer, Short-haired	24	15	39	69.2%	3.6	49.50	dolichocephalic
German Pointer, Wire-haired	26	14	40	55.0%	3.9	48.92	dolichocephalic

Giant Schnauzer	74	59	131	63.4%	3.7	47.45	dolichocephalic
Golden Retriever	100	100	200	65.5%	3.8	55.51	mesocephalic
Gordon Setter	11	19	30	60.0%	3.4	49.60	dolichocephalic
Great Dane	102	52	152	57.2%	3.0	56.59	mesocephalic
Greyhound	2	5	6	33.3%	5.7	46.34	dolichocephalic
Havanese	103	37	137	55.5%	2.0	66.52	brachycephalic
Hungarian Greyhound	3	2	4	75.0%	2.1	46.72	dolichocephalic
Irish Setter	39	28	66	42.4%	4.2	43.56	dolichocephalic
Italian Greyhound	1	0	1	100.0%	0.8	53.74	mesocephalic
Jack Russell Terrier (Russel Terrier)	100	100	200	65.5%	3.0	59.98	mesocephalic
Japanese Spitz	0	1	1	0.0%	2.0	70.96	brachycephalic
Keeshond (Wolfspitz)	14	7	21	71.4%	5.2	58.32	mesocephalic
King Charles Spaniel	3	0	3	100.0%	10.0	66.86	brachycephalic
Labrador Retriever	100	100	200	72.5%	2.4	54.85	mesocephalic
Lagotto Romagnolo	4	4	8	50.0%	1.7	58.55	mesocephalic
Leonberger	22	13	35	60.0%	2.9	53.79	mesocephalic
Maltese	62	42	102	54.9%	3.9	67.06	brachycephalic
Miniature Bull Terrier	11	20	29	62.1%	2.1	50.00	dolichocephalic
Miniature German Spitz	8	5	13	69.2%	3.6	67.67	brachycephalic
Miniature Poodle	31	14	45	55.6%	4.1	54.32	mesocephalic
Miniature Schnauzer	18	19	36	52.8%	4.3	54.20	mesocephalic
Mudi	6	1	7	71.4%	4.1	57.21	mesocephalic
Neapolitan Mastiff (Mastino Napoletano)	2	6	8	62.5%	4.4	72.56	brachycephalic
Old English Sheepdog (Bobtail)	12	17	27	55.6%	4.3	58.30	mesocephalic
Papillon (Continental Toy Spaniel)	7	11	18	44.4%	2.9	70.63	brachycephalic
Pekingese	21	9	28	53.6%	3.5	99.69	brachycephalic
Pomeranian	2	1	3	33.3%	0.7	75.68	brachycephalic
Pug	102	65	165	53.3%	2.0	101.52	brachycephalic
Puli	2	4	6	33.3%	1.6	57.74	mesocephalic
Saluki	4	6	10	60.0%	3.4	45.86	dolichocephalic
Samoyed	4	5	9	33.3%	4.2	60.42	mesocephalic
Scottish Terrier	10	6	15	33.3%	4.3	45.95	dolichocephalic
Shar Pei	24	13	37	64.9%	1.7	70.49	brachycephalic
Shiba Inu	46	11	55	41.8%	2.8	58.99	mesocephalic
Shih Tzu	41	33	74	64.9%	3.0	82.05	brachycephalic
Siberian Husky	63	59	121	55.4%	4.4	54.71	mesocephalic
Small Münsterländer	90	34	123	55.3%	4.1	48.87	dolichocephalic
Spanish Greyhound (Galgo Español)	30	26	55	61.8%	4.0	42.86	dolichocephalic
Staffordshire Bull Terrier	16	4	20	35.0%	5.8	75.78	brachycephalic
Standard Schnauzer	18	14	31	61.3%	3.7	53.64	mesocephalic
Tibetan Spaniel	1	4	5	80.0%	2.7	84.55	brachycephalic
Tibetan Terrier	58	84	139	59.0%	3.3	58.58	mesocephalic
Transylvanian Hound	0	1	1	100.0%	5.0	49.44	dolichocephalic
Vizsla, Wire-haired	2	1	2	50.0%	5.5	45.16	dolichocephalic
Weimaraner	52	45	95	56.8%	2.6	49.95	dolichocephalic
Welsh Corgi - Pembroke	1	1	1	100.0%	7.0	69.80	brachycephalic
White Swiss Shepherd Dog (Berger Blanc Suisse)	99	55	152	50.7%	3.0	46.94	dolichocephalic
Yorkshire Terrier	101	70	170	52.9%	4.0	73.01	brachycephalic

Supplementary Table S2. The four personality traits (from Survey 1) and the four behavioural problems (from Survey 2). For the personality traits, the individual items making up the trait and the internal consistency of the trait (Cronbach's alpha) is also provided. Asterix marks reverse coded items.

**Calmness**	**Trainability**
Is calm, even in ambiguous situations	Is ingenious, inventive when seeks hidden food or toy
Can be stressed easily*	Is intelligent, learns quickly
Is emotionally balanced, not easy to rile	Is very easy to warm up to a new toy
Is cool-headed even in stressful situations	Is not much interested except in eating and sleeping*
Is sometimes anxious and uncertain*	Often does not understand what was expected from him/her during playing*
Cronbach's alpha: 0.849	Cronbach's alpha: 0.711
**Dog sociability**	**Boldness**
Fights with conspecifics frequently*	Is rather cool, reserved*
Is ready to share toys with conspecifics	Is unassertive, aloof when unfamiliar persons enter the home*
Is bullying with conspecifics*	Is sometimes fearful, awkward*
Gets on well with conspecifics	
Cronbach's alpha: 0.738	Cronbach's alpha: 0.642

**Behavioural problems**	
Pulling the leash	My dog frequently pulls on the leash. I have tried everything but cannot control him/her.
Jumping up	When I arrive home my dog jumps up at me. I do not like it but (s)he does not give up.
Too reactive when guests arrive	When a visitor rings the bell my dog knows that (s)he is to stay back from the door.*
Not coming back	My dog usually does not listen to me when I call him/her back. I get really angry about it.

Supplementary Table S3. Baseline behavioural differences across head shape groups when large-sized brachycephalic dogs are excluded from the sample. Generalized linear models (GLMs) were conducted for each behavioural variable using linear or binomial logistic distributions, depending on the variable, with head shape as the sole fixed factor. Parameter estimates for the pairwise comparisons are also presented. Significant differences are marked in italics.

**Global head shape effect**			**Pairwise comparisons**					
**Behaviour**	**Wald χ^2**	**P value**	**Dog group**	**B**	**SE**	**Wald χ^2**	**P value**	**Exp(B) (95% Conf Int)**
Calmness	*12.088*	*0.002*						
			Brachy > Dolicho	*-0.157*	*0.052*	*9.316*	*0.002*	*0.854 (0.772-0.945)*
			Brachy vs. Meso	-0.018	0.046	0.151	0.698	0.982 (0.898-1.074)
			Dolicho < Meso	*0.140*	*0.045*	*9.563*	*0.002*	*1.150 (1.053-1.256)*
Trainability	*33.383*	*< 0.001*						
			Brachy < Dolicho	*0.277*	*0.052*	*28.523*	*< 0.001*	*1.319 (1.191-1.460)*
			Brachy < Meso	*0.246*	*0.049*	*25.701*	*< 0.001*	*1.279 (1.163-1.407)*
			Dolicho vs. Meso	-0.031	0.043	0.502	0.479	0.970 (0.891-1.056)
Dog sociability	0.701	0.704						
			Brachy vs. Dolicho	0.036	0.051	0.488	0.485	1.036 (0.938-1.145)
			Brachy vs. Meso	0.037	0.047	0.629	0.428	1.038 (0.947-1.138)
			Dolicho vs. Meso	0.002	0.043	0.001	0.971	1.002 (0.920-1.090)
Boldness	*8.658*	*0.013*						
			Brachy vs Dolicho	-0.095	0.052	3.343	0.067	0.909 (0.821-1.007)
			Brachy vs. Meso	0.036	0.046	0.596	0.440	1.037 (0.946-1.135)
			Dolicho < Meso	*0.131*	*0.045*	*8.583*	*0.003*	*1.140 (1.044-1.244)*
Jumping up	3.407	0.182						
			Brachy vs. Dolicho	-0.215	0.124	2.998	0.083	0.807 (0.633-1.029)
			Brachy vs. Meso	-0.169	0.110	2.367	0.124	0.844 (0.681-1.047)
			Dolicho vs. Meso	0.046	0.107	0.182	0.669	1.047 (0.849-1.290)
Pulling the leash	2.204	0.332						
			Brachy vs. Dolicho	0.058	0.125	0.217	0.642	1.060 (0.830-1.353)
			Brachy vs. Meso	-0.095	0.112	0.709	0.400	0.910 (0.730-1.134)
			Dolicho vs. Meso	-0.153	0.106	2.074	0.150	0.858 (0.697-1.057)
Too reactive	*28.346*	*< 0.001*						
			Brachy > Dolicho	*-0.819*	*0.155*	*27.93*	*< 0.001*	*0.441 (0.325-0.597)*
			Brachy > Meso	*-0.473*	*0.146*	*10.472*	*0.001*	*0.623 (0.468-0.830)*
			Dolicho < Meso	*0.346*	*0.117*	*8.749*	*0.003*	*1.414 (1.124-1.778)*
Not coming back	*15.204*	*< 0.001*						
			Brachy > Dolicho	*-0.448*	*0.119*	*14.199*	*< 0.001*	*0.639 (0.506-0.807)*
			Brachy vs. Meso	-0.162	0.107	2.310	0.129	0.850 (0.690-1.048)
			Dolicho < Meso	*0.286*	*0.100*	*8.194*	*0.004*	*1.331 (1.094-1.618)*

Supplementary Table S4. Comparison of demographic and dog keeping factors between head shape groups when large-sized brachycephalic dogs are excluded from the sample. For each categorical variable, the proportion of dogs in each category is presented separately for each head shape group. Significant group differences, as indicated by the Chi-squared tests with z post-hoc tests, are highlighted by marking the category with the larger proportion in each group in bold. Only differences with a p-value < 0.00037 are considered significant and are marked in italics. For pairwise significant differences, the absolute magnitude of the difference is provided as a measure of effect size. B: brachycephalic; M: mesocephalic; D: dolichocephalic.

Factors in both surveys	Categories	B (N = 1,256)	M (N = 2,388)	D (N = 1,439)	Statistics	Absolute magnitude of the differences
Dogs' age in years (mean ±SD)	–	2.72 (±2.74)	3.23 (±2.91)	3.68 (±3.18)	*H = 82.712* *p < 0.001*	B vs. M: 0.51 years; B vs. D: 0.96 years; M vs. D: 0.45 years
Dogs' weight in kg (mean ±SD)	–	7.27 (±5.87)	24.72 (±15.70)	27.63 (±9.29)	*H =2146.184* *p < 0.001*	B vs. M: 24.41 kg; B vs. D: 31.11 kg; M vs. D: 6.70 kg
Dogs' height in cm (mean ±SD)*	–	26.71 (±5.58)	51.12 (±13.63)	57.82 (±10.66)	*H = 1996.668* *p < 0.001*	B vs. M: 17.45 cm; B vs. D: 20.36 cm; M vs. D: 2.91 cm
Dogs' sex	Male	57.4%	59.5%	56.8%	$\chi^2 = 3.042$	
	Female	42.6%	40.5%	43.2%	p = 0.219	
Dogs' neuter status	Intact	**77.9%**	66.5%	69.5%	$\chi^2 = 51.546$	B vs. M: 11.41%; B vs. D: 8.37%
	Neutered	22.1%	**33.5%**	**30.5%**	*p < 0.001*	
Dogs' age at acquisition	bred by owner	1.3%	1.8%	2.5%	$\chi^2 = 73.829$	
	2–12 weeks	**65.2%**	**66.7%**	58.6%	*p < 0.001*	B vs. M: 6.62%; M vs. D: 8.08%
	3-12 months	**25.1%**	18.8%	21.3%		B vs. M: 6.28%
	> 1 year	8.4%	**12.7%**	**17.6%**		B vs. M: 4.29%; B vs. D: 9.14%; M vs. D: 4.85%
Dogs' training experience	no training	**53.9%**	28.4%	28.8%	$\chi^2 = 348.737$	B vs. M: 25.47%; B vs. D: 25.13%
	1 type	24.2%	23.6%	**27.7%**	*p < 0.001*	M vs. D: 4.04%
	2 types	15.4%	**24.2%**	**21.7%**		B vs. M: 8.80%; B vs. D: 6.32%
	3 or more types	6.5%	**23.8%**	**21.9%**		B vs. M: 17.26%; B vs. D: 15.36%
Owners' sex	man	14.3%	**19.3%**	**23.3%**	$\chi^2 = 35.168$	B vs. M: 4.93%; B vs. D: 9.02%; M vs. D: 4.09%
	woman	**85.7%**	**80.7%**	76.7%	*p < 0.001*	
Owners' age	≤ 18 years	5.9%	5.1%	3.5%	$\chi^2 = 54.430$	
	19-30 years	**34.2%**	27.1%	23.8%	*p < 0.001*	B vs. M: 7.10%; B vs. D: 10.32%
	31-60 years	56.9%	**65.4%**	**69.4%**		B vs. M: 8.44%; B vs. D: 12.43%; M vs. D: 3.99%
	> 60 years	3.0%	2.5%	3.3%		
Owners' education	primary school	22.7%	21.3%	20.7%	$\chi^2 = 21.873$	
	secondary school	39.5%	42.3%	39.7%	p = 0.001	
	college	28.1%	24.6%	24.9%		
	university	9.7%	11.7%	14.7%		

N of previous dogs	no previous dog	**50.0%**	**44.8%**	33.6%	$\chi^2 = 87.849$	B vs. M: 5.23%; B vs. D: 16.41%; M vs. D: 11.18%
	1 dog	23.2%	25.7%	**27.5%**	$p < 0.001$	B vs. D: 4.33%
	2 dogs	12.4%	14.4%	**18.5%**		B vs. D: 6.14%; M vs. D: 4.09%
	3 or more dogs	14.4%	15.1%	**20.4%**		B vs. D: 5.94%; M vs. D: 5.26%
Purpose of keeping the dog	family member only	**59.9%**	**43.8%**	38.8%	$\chi^2 = 133.686$	B vs. M: 16.03%; B vs. D: 21.10%; M vs. D: 5.07%
	family member + other	35.4%	**48.7%**	**52.3%**	$p < 0.001$	B vs. M: 13.31%; B vs. D: 16.90%
	not family member	4.7%	7.4%	**8.9%**		B vs. D: 4.20%
N of people in the household	only 1 person	**12.9%**	9.6%	10.8%	$\chi^2 = 23.519$	B vs. M: 3.33%
	2 people	46.7%	42.6%	45.9%	$p < 0.001$	
	3 or more people	40.4%	**47.8%**	43.3%		B vs. M: 7.45%; M vs. D: 4.52%
N of dogs in the household	no other dog	66.4%	68.3%	63.4%	$\chi^2 = 11.855$	
	1 other dog	20.7%	19.4%	23.8%	$p = 0.018$	
	≥ 2 other dogs	12.9%	12.3%	12.7%		
**Factors only in Survey 1**	**Categories**	**B (N = 741)**	**M (N = 1.281)**	**D (N = 833)**	**Statistics**	**Absolute magnitude of the differences**
Hours spent with the dog / day	≤ 3 hours	15.5%	**27.3%**	**27.4%**	$\chi^2 = 41.718$	B vs. M: 11.80%; B vs. D: 11.85%
	> 3 hours	**84.5%**	72.7%	72.6%	$p < 0.001$	
Frequency of playing / week	1-5 days	14.3%	**21.5%**	**19.0%**	$\chi^2 = 16.050$	B vs. M: 7.24%; B vs. D: 4.66%
	6-7 days	**85.7%**	78.5%	81.0%	$p < 0.001$	
**Factors only in Survey 2**	**Categories**	**B (N = 536)**	**M (N = 1.124)**	**D (N = 630)**	**Statistics**	**Absolute magnitude of the differences**
N of children in the household	1 or more	80.2%	76.0%	82.0%	$\chi^2 = 9.582$	
	None	19.8%	24.0%	18.0%	$p = 0.008$	
Where the dog is kept	only indoors	**84.7%**	68.3%	71.9%	$\chi^2 = 51.023$	B vs. M: 16.37%; B vs. D: 12.80%
	in- and outdoors	14.4%	**28.8%**	**25.2%**	$p < 0.001$	B vs. M: 14.46%; B vs. D: 10.87%
	only outdoors	0.9%	2.8%	2.9%		
Hours spend walking the dog	< 1 hour	**24.6%**	10.1%	9.2%	$\chi^2 = 89.256$	B vs. M: 14.48%; B vs. D: 15.42%
	1-3 hours	69.4%	**77.2%**	**81.0%**	$p < 0.001$	B vs. M: 7.82%; B vs. D: 11.55%
	> 3 hours	6.0%	**12.6%**	**9.8%**		B vs. M: 6.66%; B vs D: 3.87%
Hours spend playing / day	≤ 1 hour	64.9%	67.8%	67.8%	$\chi^2 = 1.524$	
	> 1 hour	35.1%	32.2%	32.2%	$p = 0.467$	
Buy gifts for the dog	Yes	26.1%	29.7%	34.8%	$\chi^2 = 10.563$	
	No	73.9%	70.3%	65.2%	$p = 0.005$	
Allow the dog into the bed	Yes	37.7%	**66.2%**	**63.7%**	$\chi^2 = 130.122$	B vs. M: 28.51%; B vs. D: 25.96%
	No	**62.3%**	33.8%	36.3%	$p < 0.001$	

* *For reference only, in the analyses, the height residual was examined instead of raw height.*

Supplementary Table S5. Associations between the potential confounding factors and behaviour. Generalized linear models (GLMs) were conducted for each association separately, using linear or binomial logistic distributions, depending on the behavioural variable. Each model included only one fixed factor (for categorical variables) or one covariate (for continuous variables). Parameter estimates for the pairwise comparisons are also presented.

Calmness

Global effect			Pairwise comparisons					
**Factor**	**Wald χ^2**	**P value**	**Categories**	**B**	**SE**	**Wald χ^2**	**P value**	**Exp(B) (95% Conf Int)**
**Dogs' age**	2.430	0.119	-	0.009	0.006	2.430	0.119	1.009 (0.998-1.021)
**Dogs' weight**	7.606	0.006	-	0.003	0.001	7.606	0.006	1.003 (1.001-1.006)
**Weight-residual of height**	8.643	0.003	-	-0.060	0.020	8.643	0.003	0.942 (0.905-0.980)
**Neuter status**	16.127	< 0.001						
reference category: neutered			intact	0.165	0.041	16.127	< 0.001	1.179 (1.088-1.278)
**Age at acquisition**	25.624	< 0.001						
reference category: > 1 year			bred by owner	0.297	0.155	3.691	0.055	1.346 (0.994-1.821)
			2–12 weeks	0.157	0.058	7.292	0.007	1.17 (1.044-1.312)
			3-12 months	-0.042	0.067	0.405	0.525	0.958 (0.841-1.092)
**Training experience**	5.442	0.142						
reference category: 3 or more types			no training	-0.097	0.052	3.538	0.060	0.907 (0.820-1.004)
			1 type	-0.073	0.055	1.743	0.187	0.930 (0.834-1.036)
			2 types	-0.008	0.058	0.017	0.896	0.992 (0.885-1.112)
**Owners' gender**	2.815	0.093						
reference category: woman			man	0.070	0.042	2.815	0.093	1.073 (0.988-1.164)
**Owners' age**	1.202	0.753						
reference category: > 60 years			≤ 18 years	-0.029	0.137	0.045	0.831	0.971 (0.742-1.271)
			19-30 years	-0.078	0.121	0.413	0.520	0.925 (0.730-1.172)
			31-60 years	-0.041	0.119	0.118	0.732	0.960 (0.761-1.212)
**Owners' education**	7.874	0.049						
reference category: university			primary school	-0.140	0.064	4.738	0.029	0.869 (0.766-0.986)
			secondary school	-0.023	0.060	0.148	0.701	0.977 (0.869-1.099)
			college	-0.033	0.062	0.276	0.600	0.968 (0.857-1.093)
**N of previous dogs**	8.227	0.042						
reference category: 3 or more dogs			no previous dog	-0.043	0.053	0.650	0.420	0.958 (0.863-1.063)
			1 dog	-0.128	0.059	4.752	0.029	0.880 (0.784-0.987)
			2 dogs	0.019	0.065	0.088	0.767	1.019 (0.898-1.158)
**Purpose of keeping the dog**	1.951	0.377						
reference category: not family member			family member only	0.074	0.079	0.857	0.355	1.076 (0.921-1.258)
			family member+other	0.103	0.080	1.680	0.195	1.109 (0.949-1.296)
**N of people in the household**	3.400	0.183						
reference category: 3 or more people			only 1 person	0.088	0.059	2.198	0.138	1.092 (0.972-1.227)
			2 people	-0.023	0.038	0.355	0.551	0.978 (0.908-1.053)
**Hours spent with the dog / day**	32.794	< 0.001						
reference category: > 3 hours			≤ 3 hours	-0.236	0.041	32.794	< 0.001	0.79 (0.728-0.856)

Trainability

Global effect			Pairwise comparisons					
**Factor**	**Wald χ^2**	**P value**	**Categories**	**B**	**SE**	**Wald χ^2**	**P value**	**Exp(B) (95% Conf Int)**
**Dogs' age**	102.787	< 0.001	-	-0.066	0.007	102.787	< 0.001	0.936 (0.924-0.948)
**Dogs' weight**	1.861	0.172	-	0.002	0.001	1.861	0.172	1.002 (0.999-1.004)

**Weight-residual of height**	37.103	< 0.001	-	0.127	0.021	37.103	< 0.001	1.136 (1.090-1.183)
**Neuter status**	14.552	< 0.001						
reference category: neutered			intact	0.162	0.042	14.552	< 0.001	1.176 (1.082-1.278)
**Age at acquisition**	85.836	< 0.001						
reference category: > 1 year			bred by owner	0.606	0.140	18.829	< 0.001	1.833 (1.394-2.411)
			2–12 weeks	0.583	0.064	83.767	< 0.001	1.791 (1.581-2.029)
			3-12 months	0.470	0.071	44.350	< 0.001	1.599 (1.393-1.836)
**Training experience**	191.255	< 0.001						
reference category: 3 or more types			no training	-0.629	0.048	171.956	< 0.001	0.533 (0.485-0.586)
			1 type	-0.327	0.049	44.305	< 0.001	0.721 (0.655-0.794)
			2 types	-0.173	0.050	12.006	0.001	0.841 (0.763-0.928)
**Owners' gender**	3.308	0.069						
reference category: woman			man	-0.078	0.043	3.308	0.069	0.925 (0.85-1.006)
**Owners' age**	1.502	0.682						
reference category: > 60 years			≤ 18 years	-0.054	0.118	0.209	0.647	0.947 (0.752-1.194)
			19-30 years	-0.033	0.096	0.119	0.730	0.967 (0.802-1.167)
			31-60 years	0.009	0.093	0.008	0.927	1.009 (0.840-1.211)
**Owners' education**	15.768	0.001						
reference category: university			primary school	-0.237	0.060	15.575	< 0.001	0.789 (0.701-0.887)
			secondary school	-0.138	0.055	6.339	0.012	0.871 (0.782-0.970)
			college	-0.123	0.058	4.590	0.032	0.884 (0.790-0.990)
**N of previous dogs**	8.308	0.040						
reference category: 3 or more dogs			no previous dog	-0.078	0.051	2.363	0.124	0.925 (0.837-1.022)
			1 dog	-0.088	0.056	2.458	0.117	0.915 (0.820-1.022)
			2 dogs	-0.070	0.063	1.227	0.268	0.932 (0.824-1.055)
**Purpose of keeping the dog**	27.333	< 0.001						
reference category: not family member			family member only	-0.228	0.073	9.715	0.002	0.796 (0.689-0.919)
			family member+other	-0.050	0.073	0.471	0.492	0.951 (0.824-1.098)
**N of people in the household**	6.060	0.048						
reference category: 3 or more people			only 1 person	0.064	0.064	1.017	0.313	1.066 (0.941-1.208)
			2 people	0.091	0.037	5.964	0.015	1.095 (1.018-1.179)
**Hours spent with the dog / day**	13.119	< 0.001						
reference category: > 3 hours			≤ 3 hours	-0.153	0.042	13.119	< 0.001	0.858 (0.790-0.932)

Dog sociability

**Global effect**			**Pairwise comparisons**					
**Factor**	**Wald χ^2**	**P value**	**Categories**	**B**	**SE**	**Wald χ^2**	**P value**	**Exp(B) (95% Conf Int)**
**Dogs' age**	184.389	< 0.001	-	-0.084	0.006	184.389	< 0.001	0.919 (0.908-0.931)
**Dogs' weight**	4.750	0.029	-	0.003	0.001	4.750	0.029	1.003 (1.000-1.005)
**Weight-residual of height**	1.241	0.265	-	0.022	0.020	1.241	0.265	1.022 (0.983-1.063)
**Neuter status**	30.080	< 0.001						
reference category: neutered			intact	0.188	0.034	30.080	< 0.001	1.207 (1.129-1.292)
**Age at acquisition**	36.608	< 0.001						
reference category: > 1 year			bred by owner	0.015	0.155	0.009	0.925	1.015 (0.748-1.376)
			2–12 weeks	0.298	0.055	29.268	< 0.001	1.347 (1.209-1.500)
			3-12 months	0.152	0.064	5.554	0.018	1.164 (1.026-1.321)
**Training experience**	31.303	< 0.001						
reference category: 3 or more types			no training	-0.102	0.053	3.753	0.053	0.903 (0.815-1.001)
			1 type	0.056	0.055	1.031	0.310	1.058 (0.949-1.178)
			2 types	0.155	0.057	7.463	0.006	1.167 (1.045-1.304)

**Owners' gender**	0.026	0.871						
reference category: woman			man	-0.007	0.042	0.026	0.871	0.993 (0.916-1.077)
**Owners' age**	5.623	0.131						
reference category: > 60 years			≤ 18 years	-0.235	0.128	3.346	0.067	0.791 (0.615-1.017)
			19-30 years	-0.092	0.106	0.743	0.389	0.912 (0.741-1.124)
			31-60 years	-0.056	0.103	0.290	0.590	0.946 (0.773-1.158)
**Owners' education**	7.217	0.065						
reference category: university			primary school	-0.043	0.065	0.444	0.505	0.957 (0.843-1.088)
			secondary school	0.080	0.060	1.730	0.188	1.083 (0.962-1.219)
			college	0.033	0.063	0.279	0.598	1.034 (0.914-1.169)
**N of previous dogs**	2.467	0.481						
reference category: 3 or more dogs			no previous dog	0.051	0.043	1.383	0.240	1.052 (0.967-1.145)
			1 dog	-0.001	0.048	0.000	0.991	0.999 (0.910-1.098)
			2 dogs	0.019	0.053	0.131	0.718	1.019 (0.919-1.131)
**Purpose of keeping the dog**	5.562	0.062						
reference category: not family member			family member only	0.183	0.081	5.142	0.023	1.201 (1.025-1.407)
			family member+other	0.188	0.081	5.396	0.020	1.207 (1.030-1.415)
**N of people in the household**	5.649	0.059						
reference category: 3 or more people			only 1 person	0.120	0.057	4.394	0.036	1.127 (1.008-1.261)
			2 people	0.065	0.038	3.001	0.083	1.067 (0.991-1.149)
**Hours spent with the dog / day**	32.664	< 0.001						
reference category: > 3 hours			≤ 3 hours	-0.242	0.042	32.664	< 0.001	0.785 (0.722-0.853)

Boldness

**Global effect**			**Pairwise comparisons**					
**Factor**	**Wald χ^2**	**P value**	**Categories**	**B**	**SE**	**Wald χ^2**	**P value**	**Exp(B) (95% Conf Int)**
**Dogs' age**	25.587	< 0.001	-	-0.030	0.006	25.587	< 0.001	0.971 (0.960-0.982)
**Dogs' weight**	0.065	0.799	-	0.000	0.001	0.065	0.799	1.000 (0.998-1.003)
**Weight-residual of height**	4.565	0.033	-	-0.045	0.021	4.565	0.033	0.956 (0.918-0.996)
**Neuter status**	17.979	< 0.001						
reference category: neutered			intact	0.145	0.034	17.979	< 0.001	1.156 (1.081-1.237)
**Age at acquisition**	58.849	< 0.001						
reference category: > 1 year			bred by owner	0.391	0.150	6.836	0.009	1.478 (1.103-1.982)
			2–12 weeks	0.399	0.058	46.563	< 0.001	1.490 (1.329-1.671)
			3-12 months	0.193	0.068	8.097	0.004	1.213 (1.062-1.385)
**Training experience**	9.746	0.021						
reference category: 3 or more types			no training	0.043	0.053	0.650	0.420	1.044 (0.941-1.158)
			1 type	0.147	0.056	6.977	0.008	1.158 (1.039-1.291)
			2 types	0.119	0.058	4.126	0.042	1.126 (1.004-1.263)
**Owners' gender**	1.575	0.209						
reference category: woman			man	0.052	0.041	1.575	0.209	1.053 (0.971-1.142)
**Owners' age**	14.173	0.003						
reference category: > 60 years			≤ 18 years	-0.258	0.120	4.632	0.031	0.773 (0.611-0.977)
			19-30 years	-0.171	0.099	2.978	0.084	0.843 (0.695-1.023)
			31-60 years	-0.278	0.097	8.274	0.004	0.757 (0.626-0.915)
**Owners' education**	0.467	0.926						
reference category: university			primary school	-0.040	0.063	0.404	0.525	0.960 (0.848-1.088)
			secondary school	-0.031	0.060	0.265	0.606	0.970 (0.862-1.090)
			college	-0.020	0.063	0.099	0.753	0.981 (0.867-1.108)
**N of previous dogs**	0.196	0.978						

reference category: 3 or more dogs			no previous dog	0.009	0.044	0.040	0.842	1.009 (0.925-1.100)
			1 dog	0.017	0.047	0.132	0.716	1.017 (0.927-1.116)
			2 dogs	0.020	0.053	0.139	0.709	1.020 (0.919-1.132)
**Purpose of keeping the dog**	4.508	0.105						
reference category: not family member			family member only	-0.104	0.070	2.222	0.136	0.901 (0.786-1.033)
			family member+other	-0.144	0.070	4.213	0.040	0.866 (0.755-0.994)
**N of people in the household**	3.555	0.169						
reference category: 3 or more people			only 1 person	-0.033	0.060	0.309	0.578	0.967 (0.861-1.087)
			2 people	0.058	0.038	2.383	0.123	1.060 (0.984-1.141)
**Hours spent with the dog / day**	0.167	0.683						
reference category: > 3 hours			≤ 3 hours	-0.017	0.041	0.167	0.683	0.983 (0.908-1.065)

Jumping up

Global effect			Pairwise comparisons					
**Factor**	**Wald χ^2**	**P value**	**Categories**	**B**	**SE**	**Wald χ^2**	**P value**	**Exp(B) (95% Conf Int)**
**Dogs' age**	60.374	< 0.001	-	-0.142	0.018	60.374	< 0.001	0.868 (0.837-0.899)
**Dogs' weight**	23.499	< 0.001	-	-0.015	0.003	23.499	< 0.001	0.985 (0.979-0.991)
**Weight-residual of height**	0.857	0.354	-	-0.042	0.045	0.857	0.354	0.959 (0.878-1.048)
**Neuter status**	36.505	< 0.001						
reference category: neutered			intact	0.473	0.078	36.505	< 0.001	1.605 (1.376-1.871)
**Age at acquisition**	12.664	0.005						
reference category: > 1 year			bred by owner	0.577	0.313	3.389	0.066	1.780 (0.963-3.290)
			2–12 weeks	0.471	0.134	12.284	< 0.001	1.601 (1.231-2.083)
			3-12 months	0.406	0.155	6.864	0.009	1.501 (1.108-2.035)
**Training experience**	38.648	< 0.001						
reference category: 3 or more types			no training	0.732	0.130	31.588	< 0.001	2.080 (1.611-2.685)
			1 type	0.768	0.136	31.835	< 0.001	2.156 (1.651-2.815)
			2 types	0.618	0.138	20.025	< 0.001	1.856 (1.416-2.434)
**Owners' gender**	1.953	0.162						
reference category: woman			man	0.159	0.114	1.953	0.162	1.172 (0.938-1.466)
**Owners' age**	5.640	0.130						
reference category: > 60 years			≤ 18 years	-0.297	0.330	0.807	0.369	0.743 (0.389-1.420)
			19-30 years	-0.277	0.268	1.071	0.301	0.758 (0.449-1.281)
			31-60 years	-0.447	0.262	2.917	0.088	0.640 (0.383-1.068)
**Owners' education**	8.825	0.032						
reference category: university			primary school	0.453	0.159	8.131	0.004	1.573 (1.152-2.147)
			secondary school	0.247	0.143	2.982	0.084	1.280 (0.967-1.695)
			college	0.335	0.153	4.790	0.029	1.399 (1.036-1.889)
**N of previous dogs**	8.714	0.033						
reference category: 3 or more dogs			no previous dog	0.221	0.106	4.306	0.038	1.247 (1.012-1.537)
			1 dog	0.249	0.114	4.748	0.029	1.283 (1.025-1.606)
			2 dogs	0.365	0.127	8.310	0.004	1.440 (1.124-1.846)
**Purpose of keeping the dog**	1.585	0.453						
reference category: not family member			family member only	0.136	0.162	0.706	0.401	1.146 (0.834-1.573)
			family member+other	0.035	0.162	0.047	0.829	1.036 (0.754-1.422)
**N of people in the household**	1.480	0.477						
reference category: 3 or more people			only 1 person	-0.172	0.147	1.372	0.242	0.842 (0.631-1.123)
			2 people	-0.007	0.091	0.006	0.940	0.993 (0.831-1.187)
**Where the dog is kept**	4.210	0.122						
reference category: only outdoors			only indoors	-0.282	0.269	1.100	0.294	0.754 (0.445-1.278)

Factor	Wald χ²	P value	Categories	B	SE	Wald χ²	P value	Exp(B) (95% Conf Int)
			in- and outdoors	-0.099	0.277	0.129	0.720	0.905 (0.526-1.559)
**Hours spend walking the dog**	8.241	0.016						
reference category: > 3 hours			< 1 hour	0.433	0.177	5.978	0.014	1.541 (1.090-2.180)
			1-3 hours	0.108	0.145	0.551	0.458	1.114 (0.838-1.480)
**Allow the dog into the bed**	1.375	0.241						
reference category: yes			no	-0.102	0.087	1.375	0.241	0.903 (0.762-1.071)

Pulling the leash

**Global effect**			**Pairwise comparisons**					
**Factor**	**Wald χ^2**	**P value**	**Categories**	**B**	**SE**	**Wald χ^2**	**P value**	**Exp(B) (95% Conf Int)**
**Dogs' age**	38.973	< 0.001	-	-0.102	0.016	38.973	< 0.001	0.903 (0.875-0.933)
**Dogs' weight**	12.947	< 0.001	-	-0.011	0.003	12.947	< 0.001	0.989 (0.984-0.995)
**Weight-residual of height**	1.637	0.201	-	0.059	0.046	1.637	0.201	1.060 (0.969-1.160)
**Neuter status**	9.366	0.002						
reference category: neutered			intact	0.240	0.078	9.366	0.002	1.271 (1.090-1.481)
**Age at acquisition**	4.699	0.195						
reference category: > 1 year			bred by owner	-0.100	0.334	0.090	0.765	0.905 (0.470-1.741)
			2–12 weeks	0.125	0.129	0.949	0.330	1.134 (0.881-1.459)
			3-12 months	0.289	0.149	3.764	0.052	1.335 (0.997-1.788)
**Training experience**	56.111	< 0.001						
reference category: 3 or more types			no training	0.917	0.136	45.327	< 0.001	2.502 (1.916-3.267)
			1 type	0.961	0.142	45.929	< 0.001	2.614 (1.980-3.451)
			2 types	0.878	0.143	37.628	< 0.001	2.407 (1.818-3.187)
**Owners' gender**	0.097	0.755						
reference category: woman			man	0.036	0.116	0.097	0.755	1.037 (0.826-1.301)
**Owners' age**	1.731	0.630						
reference category: > 60 years			≤ 18 years	0.059	0.335	0.031	0.860	1.061 (0.550-2.046)
			19-30 years	-0.106	0.276	0.148	0.701	0.899 (0.524-1.545)
			31-60 years	-0.171	0.270	0.403	0.526	0.843 (0.497-1.430)
**Owners' education**	17.408	0.001						
reference category: university			primary school	0.658	0.162	16.555	< 0.001	1.931 (1.406-2.652)
			secondary school	0.347	0.147	5.565	0.018	1.415 (1.060-1.888)
			college	0.411	0.157	6.827	0.009	1.508 (1.108-2.053)
**N of previous dogs**	24.486	< 0.001						
reference category: 3 or more dogs			no previous dog	0.517	0.112	21.472	< 0.001	1.677 (1.347-2.086)
			1 dog	0.424	0.120	12.487	< 0.001	1.528 (1.208-1.933)
			2 dogs	0.230	0.135	2.890	0.089	1.259 (0.965-1.641)
**Purpose of keeping the dog**	19.182	< 0.001						
reference category: not family member			family member only	0.434	0.167	6.754	0.009	1.543 (1.113-2.141)
			family member+other	0.068	0.168	0.163	0.686	1.070 (0.770-1.488)
**N of people in the household**	3.365	0.186						
reference category: 3 or more people			only 1 person	-0.269	0.150	3.228	0.072	0.764 (0.569-1.025)
			2 people	-0.021	0.091	0.053	0.817	0.979 (0.819-1.170)
**Where the dog is kept**	0.720	0.698						
reference category: only outdoors			only indoors	0.182	0.290	0.394	0.530	1.200 (0.680-2.118)
			in- and outdoors	0.233	0.298	0.612	0.434	1.263 (0.704-2.266)
**Hours spend walking the dog**	12.196	0.002						
reference category: > 3 hours			< 1 hour	0.580	0.180	10.368	0.001	1.785 (1.255-2.540)
			1-3 hours	0.218	0.149	2.135	0.144	1.243 (0.928-1.665)
**Allow the dog into the bed**	1.089	0.297						

reference category: yes			no	-0.091	0.087	1.089	0.297	0.913 (0.769-1.083)

Too reactive when guests arrive

**Global effect**			**Pairwise comparisons**					
**Factor**	**Wald χ^2**	**P value**	**Categories**	**B**	**SE**	**Wald χ^2**	**P value**	**Exp(B) (95% Conf Int)**
**Dogs' age**	2.356	0.125	-	-0.025	0.016	2.356	0.125	0.975 (0.945-1.007)
**Dogs' weight**	23.342	< 0.001	-	-0.015	0.003	23.342	< 0.001	0.985 (0.979-0.991)
**Weight-residual of height**	11.013	0.001	-	-0.212	0.064	11.013	0.001	0.809 (0.713-0.917)
**Neuter status**	1.415	0.234						
reference category: neutered			intact	0.106	0.089	1.415	0.234	1.111 (0.934-1.323)
**Age at acquisition**	11.616	0.009						
reference category: > 1 year			bred by owner	-0.100	0.336	0.089	0.766	0.905 (0.469-1.747)
			2–12 weeks	0.392	0.139	7.983	0.005	1.479 (1.127-1.941)
			3-12 months	0.123	0.161	0.582	0.445	1.131 (0.824-1.552)
**Training experience**	4.636	0.200						
reference category: 3 or more types			no training	0.192	0.136	1.991	0.158	1.212 (0.928-1.582)
			1 type	0.232	0.145	2.560	0.110	1.262 (0.949-1.677)
			2 types	0.301	0.148	4.150	0.042	1.352 (1.011-1.807)
**Owners' gender**	1.511	0.219						
reference category: woman			man	-0.160	0.131	1.511	0.219	0.852 (0.659-1.100)
**Owners' age**	15.447	0.001						
reference category: > 60 years			≤ 18 years	0.163	0.417	0.152	0.696	1.176 (0.520-2.662)
			19-30 years	0.222	0.337	0.434	0.510	1.248 (0.645-2.414)
			31-60 years	-0.224	0.326	0.472	0.492	0.799 (0.422-1.515)
**Owners' education**	3.453	0.327						
reference category: university			primary school	0.112	0.186	0.364	0.546	1.119 (0.777-1.611)
			secondary school	-0.134	0.161	0.699	0.403	0.874 (0.638-1.198)
			college	-0.106	0.173	0.372	0.542	0.900 (0.640-1.264)
**N of previous dogs**	20.592	< 0.001						
reference category: 3 or more dogs			no previous dog	0.530	0.118	20.286	< 0.001	1.699 (1.349-2.139)
			1 dog	0.292	0.125	5.425	0.020	1.339 (1.047-1.711)
			2 dogs	0.328	0.143	5.294	0.021	1.389 (1.050-1.837)
**Purpose of keeping the dog**	3.443	0.179						
reference category: not family member			family member only	0.319	0.177	3.265	0.071	1.376 (0.973-1.946)
			family member+other	0.219	0.175	1.558	0.212	1.245 (0.883-1.755)
**N of people in the household**	10.704	0.005						
reference category: 3 or more people			only 1 person	0.153	0.165	0.865	0.352	1.166 (0.844-1.610)
			2 people	0.346	0.106	10.696	0.001	1.414 (1.149-1.740)
**Where the dog is kept**	6.538	0.038						
reference category: only outdoors			only indoors	0.082	0.310	0.070	0.791	1.085 (0.591-1.992)
			in- and outdoors	0.389	0.323	1.447	0.229	1.475 (0.783-2.778)
**Hours spend walking the dog**	14.839	0.001						
reference category: > 3 hours			< 1 hour	0.781	0.220	12.587	< 0.001	2.184 (1.419-3.363)
			1-3 hours	0.158	0.156	1.023	0.312	1.171 (0.862-1.590)
**Allow the dog into the bed**	18.499	< 0.001						
reference category: yes			no	-0.453	0.105	18.499	< 0.001	0.636 (0.517-0.782)

Not coming back

**Global effect**			**Pairwise comparisons**					
**Factor**	**Wald χ^2**	**P value**	**Categories**	**B**	**SE**	**Wald χ^2**	**P value**	**Exp(B) (95% Conf Int)**

**Dogs' age**	34.665	< 0.001	-	-0.084	0.014	34.665	< 0.001	0.919 (0.894-0.945)
**Dogs' weight**	11.658	0.001	-	-0.009	0.003	11.658	0.001	0.991 (0.985-0.996)
**Weight-residual of height**	7.542	0.006	-	-0.127	0.046	7.542	0.006	0.881 (0.804-0.964)
**Neuter status**	3.583	0.058						
reference category: neutered			intact	0.135	0.071	3.583	0.058	1.144 (0.995-1.315)
**Age at acquisition**	2.893	0.408						
reference category: > 1 year			bred by owner	-0.378	0.299	1.602	0.206	0.685 (0.381-1.231)
			2–12 weeks	0.077	0.118	0.422	0.516	1.080 (0.857-1.360)
			3-12 months	0.072	0.138	0.274	0.601	1.075 (0.820-1.410)
**Training experience**	58.087	< 0.001						
reference category: 3 or more types			no training	0.725	0.115	39.835	< 0.001	2.066 (1.649-2.587)
			1 type	0.832	0.122	46.469	< 0.001	2.297 (1.809-2.918)
			2 types	0.722	0.122	34.878	< 0.001	2.059 (1.620-2.617)
**Owners' gender**	2.533	0.111						
reference category: woman			man	0.175	0.110	2.533	0.111	1.191 (0.960-1.477)
**Owners' age**	31.825	< 0.001						
reference category: > 60 years			≤ 18 years	1.030	0.342	9.090	0.003	2.800 (1.434-5.468)
			19-30 years	0.316	0.263	1.438	0.230	1.372 (0.818-2.298)
			31-60 years	-0.034	0.257	0.018	0.894	0.966 (0.584-1.599)
**Owners' education**	8.704	0.033						
reference category: university			primary school	0.427	0.146	8.563	0.003	1.533 (1.151-2.040)
			secondary school	0.288	0.128	5.046	0.025	1.334 (1.037-1.715)
			college	0.262	0.139	3.557	0.059	1.299 (0.990-1.705)
**N of previous dogs**	75.844	< 0.001						
reference category: 3 or more dogs			no previous dog	0.833	0.100	69.592	< 0.001	2.300 (1.891-2.797)
			1 dog	0.663	0.107	38.095	< 0.001	1.940 (1.572-2.395)
			2 dogs	0.402	0.120	11.267	0.001	1.495 (1.182-1.891)
**Purpose of keeping the dog**	5.198	0.074						
reference category: not family member			family member only	-0.058	0.152	0.143	0.705	0.944 (0.701-1.272)
			family member+other	-0.230	0.151	2.303	0.129	0.795 (0.591-1.069)
**N of people in the household**	5.751	0.056						
reference category: 3 or more people			only 1 person	-0.253	0.135	3.493	0.062	0.777 (0.596-1.012)
			2 people	-0.176	0.086	4.215	0.040	0.838 (0.708-0.992)
**Where the dog is kept**	1.072	0.585						
reference category: only outdoors			only indoors	-0.249	0.266	0.877	0.349	0.779 (0.462-1.313)
			in- and outdoors	-0.200	0.274	0.532	0.466	0.819 (0.478-1.402)
**Hours spend walking the dog**	22.188	< 0.001						
reference category: > 3 hours			< 1 hour	0.772	0.170	20.632	< 0.001	2.165 (1.551-3.022)
			1-3 hours	0.295	0.133	4.936	0.026	1.344 (1.035-1.744)
**Allow the dog into the bed**	0.046	0.830						
reference category: yes			no	0.018	0.082	0.046	0.830	1.018 (0.867-1.195)

Supplementary Table S6. Associations between the potential confounding factors and behaviour when large-sized brachycephalic dogs are excluded from the sample. Generalized linear models (GLMs) were conducted for each association separately, using linear or binomial logistic distributions, depending on the behavioural variable. Each model included only one fixed factor (for categorical variables) or one covariate (for continuous variables). Parameter estimates for the pairwise comparisons are also presented.

**Calmness**								
**Global effect**			**Pairwise comparisons**					
**Factor**	**Wald χ^2**	**P value**	**Categories**	**B**	**SE**	**Wald χ^2**	**P value**	**Exp(B) (95% Conf Int)**
**Dogs' age**	1.507	0.220	-	0.008	0.006	1.507	0.220	1.008 (0.995-1.020)
**Dogs' weight**	4.651	0.031	-	0.003	0.001	4.651	0.031	1.003 (1.000-1.005)
**Weight-residual of height**	4.540	0.033	-	-0.048	0.022	4.540	0.033	0.953 (0.913-0.996)
**Neuter status**	14.072	< 0.001						
reference category: neutered			intact	0.164	0.044	14.072	< 0.001	1.178 (1.081-1.283)
**Age at acquisition**	26.498	< 0.001						
reference category: > 1 year			bred by owner	0.320	0.167	3.686	0.055	1.377 (0.993-1.909)
			2–12 weeks	0.210	0.063	11.071	0.001	1.233 (1.090-1.395)
			3-12 months	0.009	0.072	0.016	0.898	1.009 (0.877-1.162)
**Training experience**	8.801	0.032						
reference category: 3 or more types			no training	-0.129	0.054	5.669	0.017	0.879 (0.791-0.977)
			1 type	-0.074	0.058	1.633	0.201	0.928 (0.828-1.040)
			2 types	-0.002	0.061	0.001	0.975	0.998 (0.886-1.125)
**Owners' gender**	1.589	0.207						
reference category: woman			man	0.056	0.045	1.589	0.207	1.058 (0.969-1.154)
**Owners' age**	1.061	0.786						
reference category: > 60 years			≤ 18 years	-0.041	0.141	0.085	0.771	0.960 (0.727-1.266)
			19-30 years	-0.100	0.124	0.652	0.419	0.905 (0.709-1.154)
			31-60 years	-0.080	0.122	0.427	0.513	0.923 (0.727-1.172)
**N of previous dogs**	6.597	0.086						
reference category: 3 or more dogs			no previous dog	-0.027	0.056	0.241	0.623	0.973 (0.872-1.086)
			1 dog	-0.115	0.062	3.439	0.064	0.891 (0.789-1.007)
			2 dogs	0.028	0.070	0.161	0.688	1.028 (0.897-1.179)
**Purpose of keeping the dog**	1.181	0.554						
reference category: not family member			family member only	0.022	0.084	0.067	0.796	1.022 (0.867-1.204)
			family member+other	0.059	0.084	0.496	0.481	1.061 (0.900-1.250)
**N of people in the household**	3.576	0.167						
reference category: 3 or more people			only 1 person	0.103	0.063	2.719	0.099	1.109 (0.981-1.254)
			2 people	-0.016	0.040	0.153	0.696	0.985 (0.911-1.064)
**Hours spent with the dog / day**	23.670	< 0.001						
reference category: > 3 hours			≤ 3 hours	-0.212	0.044	23.670	< 0.001	0.809 (0.743-0.881)
**Frequency of playing / week**	19.790	< 0.001						
reference category: 6-7 days			1-5 days	-0.217	0.049	19.790	< 0.001	0.805 (0.731-0.886)

**Trainability**								
**Global effect**			**Pairwise comparisons**					
**Factor**	**Wald χ^2**	**P value**	**Categories**	**B**	**SE**	**Wald χ^2**	**P value**	**Exp(B) (95% Conf Int)**
**Dogs' age**	96.323	< 0.001	-	-0.069	0.007	96.323	< 0.001	0.934 (0.921-0.946)
**Dogs' weight**	1.864	0.172	-	0.002	0.001	1.864	0.172	1.002 (0.999-1.004)
**Weight-residual of height**	37.812	< 0.001	-	0.142	0.023	37.812	< 0.001	1.153 (1.102-1.206)

**Neuter status**	11.453	0.001						
reference category: neutered			intact	0.155	0.046	11.453	0.001	1.167 (1.067-1.276)
**Age at acquisition**	86.001	< 0.001						
reference category: > 1 year			bred by owner	0.722	0.153	22.402	< 0.001	2.059 (1.527-2.776)
			2–12 weeks	0.631	0.070	82.195	< 0.001	1.88 (1.640-2.155)
			3-12 months	0.500	0.077	42.126	< 0.001	1.649 (1.418-1.918)
**Training experience**	211.375	< 0.001						
reference category: 3 or more types			no training	-0.691	0.050	192.900	< 0.001	0.501 (0.455-0.552)
			1 type	-0.359	0.051	48.890	< 0.001	0.699 (0.632-0.772)
			2 types	-0.188	0.052	13.232	< 0.001	0.829 (0.749-0.917)
**Owners' gender**	4.009	0.045						
reference category: woman			man	-0.094	0.047	4.009	0.045	0.911 (0.831-0.998)
**Owners' age**	2.015	0.569						
reference category: > 60 years			≤ 18 years	-0.031	0.123	0.062	0.803	0.970 (0.762-1.235)
			19-30 years	-0.024	0.102	0.055	0.814	0.976 (0.800-1.192)
			31-60 years	0.032	0.098	0.109	0.742	1.033 (0.852-1.252)
**N of previous dogs**	2.357	0.502						
reference category: 3 or more dogs			no previous dog	-0.076	0.054	1.937	0.164	0.927 (0.834-1.031)
			1 dog	-0.084	0.060	1.932	0.165	0.919 (0.817-1.035)
			2 dogs	-0.056	0.068	0.675	0.411	0.945 (0.827-1.081)
**Purpose of keeping the dog**	27.835	< 0.001						
reference category: not family member			family member only	-0.232	0.081	8.266	0.004	0.793 (0.676-0.929)
			family member+other	-0.037	0.081	0.215	0.643	0.963 (0.822-1.128)
**N of people in the household**	5.755	0.056						
reference category: 3 or more people			only 1 person	0.049	0.068	0.531	0.466	1.051 (0.920-1.199)
			2 people	0.096	0.040	5.754	0.016	1.100 (1.018-1.190)
**Hours spent with the dog / day**	12.382	< 0.001						
reference category: > 3 hours			≤ 3 hours	-0.157	0.045	12.382	< 0.001	0.854 (0.783-0.933)
**Frequency of playing / week**	83.155	< 0.001						
reference category: 6-7 days			1-5 days	-0.489	0.054	83.155	< 0.001	0.614 (0.552-0.681)

Dog sociability

**Global effect**			**Pairwise comparisons**					
**Factor**	**Wald χ^2**	**P value**	**Categories**	**B**	**SE**	**Wald χ^2**	**P value**	**Exp(B) (95% Conf Int)**
**Dogs' age**	150.950	< 0.001	-	-0.081	0.007	150.950	< 0.001	0.923 (0.911-0.935)
**Dogs' weight**	6.263	0.012	-	0.003	0.001	6.263	0.012	1.003 (1.001-1.006)
**Weight-residual of height**	0.261	0.609	-	0.011	0.021	0.261	0.609	1.011 (0.969-1.054)
**Neuter status**	18.293	< 0.001						
reference category: neutered			intact	0.181	0.042	18.293	< 0.001	1.198 (1.103-1.302)
**Age at acquisition**	41.445	< 0.001						
reference category: > 1 year			bred by owner	-0.020	0.176	0.013	0.910	0.980 (0.694-1.385)
			2–12 weeks	0.338	0.060	31.602	< 0.001	1.402 (1.246-1.578)
			3-12 months	0.171	0.070	6.018	0.014	1.186 (1.035-1.360)
**Training experience**	39.078	< 0.001						
reference category: 3 or more types			no training	-0.155	0.054	8.185	0.004	0.856 (0.770-0.952)
			1 type	0.043	0.057	0.582	0.445	1.044 (0.934-1.167)
			2 types	0.141	0.058	5.894	0.015	1.152 (1.028-1.291)
**Owners' gender**	0.019	0.890						
reference category: woman			man	-0.006	0.044	0.019	0.890	0.994 (0.912-1.083)
**Owners' age**	6.039	0.110						

reference category: > 60 years			≤ 18 years	-0.266	0.129	4.268	0.039	0.766 (0.595-0.986)
			19-30 years	-0.124	0.105	1.411	0.235	0.883 (0.719-1.084)
			31-60 years	-0.082	0.101	0.666	0.415	0.921 (0.756-1.122)
**N of previous dogs**	6.269	0.099						
reference category: 3 or more dogs			no previous dog	0.095	0.054	3.100	0.078	1.100 (0.989-1.223)
			1 dog	0.006	0.061	0.009	0.926	1.006 (0.892-1.134)
			2 dogs	0.107	0.066	2.654	0.103	1.113 (0.978-1.267)
**Purpose of keeping the dog**	6.752	0.034						
reference category: not family member			family member only	0.209	0.087	5.793	0.016	1.232 (1.039-1.460)
			family member+other	0.225	0.087	6.732	0.009	1.253 (1.057-1.485)
**N of people in the household**	5.375	0.068						
reference category: 3 or more people			only 1 person	0.119	0.060	3.877	0.049	1.126 (1.001-1.268)
			2 people	0.070	0.039	3.180	0.075	1.073 (0.993-1.159)
**Hours spent with the dog / day**	24.247	< 0.001						
reference category: > 3 hours			≤ 3 hours	-0.217	0.044	24.247	< 0.001	0.805 (0.739-0.878)
**Frequency of playing / week**	49.473	< 0.001						
reference category: 6-7 days			1-5 days	-0.345	0.049	49.473	< 0.001	0.708 (0.643-0.780)

Boldness

**Global effect**			**Pairwise comparisons**					
**Factor**	**Wald χ^2**	**P value**	**Categories**	**B**	**SE**	**Wald χ^2**	**P value**	**Exp(B) (95% Conf Int)**
**Dogs' age**	30.541	< 0.001	-	-0.035	0.006	30.541	< 0.001	0.966 (0.954-0.978)
**Dogs' weight**	0.015	0.901	-	0.000	0.001	0.015	0.901	1.000 (0.998-1.003)
**Weight-residual of height**	5.754	0.016	-	-0.055	0.023	5.754	0.016	0.947 (0.905-0.990)
**Neuter status**	24.599	< 0.001						
reference category: neutered			intact	0.216	0.044	24.599	< 0.001	1.242 (1.14-1.353)
**Age at acquisition**	50.229	< 0.001						
reference category: > 1 year			bred by owner	0.408	0.157	6.794	0.009	1.504 (1.107-2.045)
			2–12 weeks	0.413	0.063	43.063	< 0.001	1.511 (1.336-1.710)
			3-12 months	0.238	0.073	10.738	0.001	1.269 (1.100-1.463)
**Training experience**	10.167	0.017						
reference category: 3 or more types			no training	0.055	0.056	0.976	0.323	1.056 (0.947-1.178)
			1 type	0.163	0.058	7.878	0.005	1.177 (1.050-1.318)
			2 types	0.129	0.061	4.409	0.036	1.137 (1.009-1.283)
**Owners' gender**	1.536	0.215						
reference category: woman			man	0.055	0.045	1.536	0.215	1.057 (0.968-1.153)
**Owners' age**	13.602	0.004						
reference category: > 60 years			≤ 18 years	-0.233	0.125	3.510	0.061	0.792 (0.620-1.011)
			19-30 years	-0.148	0.105	2.009	0.156	0.862 (0.702-1.058)
			31-60 years	-0.269	0.102	6.974	0.008	0.764 (0.626-0.933)
**N of previous dogs**	0.294	0.961						
reference category: 3 or more dogs			no previous dog	-0.014	0.054	0.063	0.801	0.986 (0.887-1.097)
			1 dog	-0.023	0.060	0.143	0.705	0.978 (0.869-1.099)
			2 dogs	0.007	0.067	0.010	0.922	1.007 (0.883-1.147)
**Purpose of keeping the dog**	3.849	0.146						
reference category: not family member			family member only	-0.106	0.075	1.994	0.158	0.899 (0.776-1.042)
			family member+other	-0.144	0.076	3.621	0.057	0.866 (0.747-1.004)
**N of people in the household**	4.758	0.093						
reference category: 3 or more people			only 1 person	-0.067	0.064	1.079	0.299	0.936 (0.825-1.061)
			2 people	0.060	0.040	2.311	0.128	1.062 (0.983-1.148)

**Hours spent with the dog / day**	0.036	0.849						
reference category: > 3 hours			≤ 3 hours	-0.008	0.043	0.036	0.849	0.992 (0.912-1.079)
**Frequency of playing / week**	1.611	0.204						
reference category: 6-7 days			1-5 days	-0.061	0.048	1.611	0.204	0.940 (0.855-1.034)

Jumping up

**Global effect**			**Pairwise comparisons**					
**Factor**	**Wald χ^2**	**P value**	**Categories**	**B**	**SE**	**Wald χ^2**	**P value**	**Exp(B) (95% Conf Int)**
**Dogs' age**	51.982	< 0.001	-	-0.137	0.019	51.982	< 0.001	0.872 (0.84 -0.905)
**Dogs' weight**	13.075	< 0.001	-	-0.012	0.003	13.075	< 0.001	0.988 (0.982-0.995)
**Weight-residual of height**	6.109	0.013	-	-0.127	0.051	6.109	0.013	0.881 (0.796-0.974)
**Neuter status**	27.338	< 0.001						
reference category: neutered			intact	0.517	0.099	27.338	< 0.001	1.677 (1.381-2.035)
**Age at acquisition**	8.296	0.040						
reference category: > 1 year			bred by owner	0.509	0.317	2.577	0.108	1.664 (0.894-3.099)
			2–12 weeks	0.396	0.141	7.945	0.005	1.486 (1.128-1.958)
			3-12 months	0.347	0.162	4.580	0.032	1.415 (1.030-1.944)
**Training experience**	37.154	< 0.001						
reference category: 3 or more types			no training	0.732	0.134	29.795	< 0.001	2.080 (1.599-2.706)
			1 type	0.780	0.140	30.912	< 0.001	2.181 (1.657-2.870)
			2 types	0.610	0.142	18.404	< 0.001	1.841 (1.393-2.433)
**Owners' gender**	2.314	0.128						
reference category: woman			man	0.181	0.119	2.314	0.128	1.199 (0.949-1.513)
**Owners' age**	5.144	0.162						
reference category: > 60 years			≤ 18 years	-0.390	0.344	1.284	0.257	0.677 (0.345-1.329)
			19-30 years	-0.300	0.282	1.130	0.288	0.741 (0.426-1.288)
			31-60 years	-0.467	0.276	2.875	0.090	0.627 (0.365-1.076)
**N of previous dogs**	3.655	0.301						
reference category: 3 or more dogs			no previous dog	0.198	0.135	2.157	0.142	1.219 (0.936-1.588)
			1 dog	0.264	0.145	3.327	0.068	1.302 (0.980-1.730)
			2 dogs	0.245	0.161	2.332	0.127	1.278 (0.933-1.751)
**Purpose of keeping the dog**	1.657	0.437						
reference category: not family member			family member only	0.155	0.167	0.861	0.353	1.167 (0.842-1.618)
			family member+other	0.051	0.167	0.095	0.758	1.053 (0.759-1.459)
**N of people in the household**	1.398	0.497						
reference category: 3 or more people			only 1 person	-0.171	0.153	1.246	0.264	0.843 (0.624-1.138)
			2 people	0.000	0.094	0.000	0.998	1.000 (0.832-1.203)
**Where the dog is kept**	3.991	0.136						
reference category: only outdoors			only indoors	-0.264	0.282	0.872	0.350	0.768 (0.442-1.336)
			in- and outdoors	-0.075	0.291	0.066	0.797	0.928 (0.524-1.641)
**Hours spend walking the dog**	6.921	0.031						
reference category: > 3 hours			< 1 hour	0.410	0.184	4.974	0.026	1.506 (1.051-2.158)
			1-3 hours	0.098	0.150	0.427	0.514	1.103 (0.822-1.481)
**Allow the dog into the bed**	1.052	0.305						
reference category: yes			no	-0.092	0.090	1.052	0.305	0.912 (0.765-1.088)

Pulling the leash

**Global effect**			**Pairwise comparisons**					
**Factor**	**Wald χ^2**	**P value**	**Categories**	**B**	**SE**	**Wald χ^2**	**P value**	**Exp(B) (95% Conf Int)**
**Dogs' age**	32.638	< 0.001	-	-0.097	0.017	32.638	< 0.001	0.907 (0.878-0.938)

**Dogs’ weight**	7.665	0.006	-	-0.009	0.003	7.665	0.006	0.991 (0.985-0.997)
**Weight-residual of height**	0.004	0.950	-	0.003	0.051	0.004	0.950	1.003 (0.907-1.110)
**Neuter status**	6.039	0.014						
reference category: neutered			intact	0.239	0.097	6.039	0.014	1.270 (1.050-1.536)
**Age at acquisition**	3.691	0.297						
reference category: > 1 year			bred by owner	-0.118	0.338	0.123	0.726	0.888 (0.458-1.722)
			2–12 weeks	0.090	0.137	0.431	0.512	1.094 (0.837-1.430)
			3-12 months	0.254	0.157	2.613	0.106	1.290 (0.947-1.756)
**Training experience**	51.474	< 0.001						
reference category: 3 or more types			no training	0.913	0.140	42.264	< 0.001	2.492 (1.892-3.281)
			1 type	0.942	0.146	41.447	< 0.001	2.566 (1.926-3.418)
			2 types	0.852	0.148	33.239	< 0.001	2.343 (1.754-3.130)
**Owners’ gender**	0.000	0.986						
reference category: woman			man	-0.002	0.122	0.000	0.986	0.998 (0.785-1.268)
**Owners’ age**	1.837	0.607						
reference category: > 60 years			≤ 18 years	0.000	0.350	0.000	1.000	1.000 (0.504-1.984)
			19-30 years	-0.103	0.291	0.125	0.723	0.902 (0.510-1.596)
			31-60 years	-0.198	0.284	0.486	0.486	0.820 (0.470-1.432)
**N of previous dogs**	12.566	0.006						
reference category: 3 or more dogs			no previous dog	0.480	0.140	11.818	0.001	1.616 (1.229-2.124)
			1 dog	0.388	0.150	6.656	0.010	1.474 (1.098-1.979)
			2 dogs	0.270	0.167	2.593	0.107	1.310 (0.943-1.818)
**Purpose of keeping the dog**	16.770	< 0.001						
reference category: not family member			family member only	0.456	0.173	6.950	0.008	1.578 (1.124-2.216)
			family member+other	0.109	0.174	0.393	0.531	1.115 (0.793-1.569)
**N of people in the household**	1.876	0.391						
reference category: 3 or more people			only 1 person	-0.205	0.156	1.732	0.188	0.815 (0.601-1.105)
			2 people	-0.007	0.095	0.005	0.944	0.993 (0.825-1.196)
**Where the dog is kept**	0.273	0.873						
reference category: only outdoors			only indoors	0.048	0.296	0.026	0.872	1.049 (0.587-1.876)
			in- and outdoors	0.098	0.305	0.103	0.749	1.103 (0.606-2.006)
**Hours spend walking the dog**	11.957	0.003						
reference category: > 3 hours			< 1 hour	-0.560	0.186	9.035	0.003	0.571 (0.397-0.823)
			1-3 hours	-0.161	0.154	1.090	0.297	0.852 (0.630-1.151)
**Allow the dog into the bed**	0.598	0.439						
reference category: yes			no	-0.070	0.091	0.598	0.439	0.932 (0.780-1.114)
**Too reactive when guests arrive**								
**Global effect**			**Pairwise comparisons**					
**Factor**	**Wald χ^2**	**P value**	**Categories**	**B**	**SE**	**Wald χ^2**	**P value**	**Exp(B) (95% Conf Int)**
**Dogs’ age**	1.346	0.246	-	-0.020	0.017	1.346	0.246	0.980 (0.948-1.014)
**Dogs’ weight**	23.704	< 0.001	-	-0.016	0.003	23.704	< 0.001	0.984 (0.977-0.990)
**Weight-residual of height**	21.703	< 0.001	-	-0.306	0.066	21.703	< 0.001	0.736 (0.647-0.838)
**Neuter status**	2.541	0.111						
reference category: neutered			intact	0.174	0.109	2.541	0.111	1.19 (0.961-1.473)
**Age at acquisition**	11.226	0.011						
reference category: > 1 year			bred by owner	-0.009	0.347	0.001	0.980	0.991 (0.502-1.957)
			2–12 weeks	0.416	0.147	7.941	0.005	1.515 (1.135-2.023)
			3-12 months	0.131	0.171	0.587	0.444	1.140 (0.816-1.593)
**Training experience**	5.312	0.150						

Factor	Wald χ^2	P value	Categories	B	SE	Wald χ^2	P value	Exp(B) (95% Conf Int)
reference category: 3 or more types			no training	0.283	0.142	3.949	0.047	1.327 (1.004-1.755)
			1 type	0.240	0.150	2.538	0.111	1.271 (0.946-1.706)
			2 types	0.304	0.153	3.950	0.047	1.355 (1.004-1.828)
**Owners' gender**	1.604	0.205						
reference category: woman			man	-0.174	0.137	1.604	0.205	0.840 (0.642-1.100)
**Owners' age**	15.734	0.001						
reference category: > 60 years			≤ 18 years	0.108	0.443	0.059	0.807	1.114 (0.467-2.656)
			19-30 years	0.168	0.365	0.211	0.646	1.183 (0.578-2.421)
			31-60 years	-0.306	0.354	0.745	0.388	0.737 (0.368-1.475)
**N of previous dogs**	15.342	0.002						
reference category: 3 or more dogs			no previous dog	0.568	0.145	15.304	< 0.001	1.765 (1.328-2.347)
			1 dog	0.387	0.156	6.143	0.013	1.473 (1.084-2.000)
			2 dogs	0.408	0.177	5.328	0.021	1.504 (1.063-2.126)
**Purpose of keeping the dog**	3.936	0.140						
reference category: not family member			family member only	0.307	0.185	2.742	0.098	1.359 (0.945-1.954)
			family member+other	0.136	0.183	0.550	0.458	1.145 (0.800-1.639)
**N of people in the household**	10.081	0.006						
reference category: 3 or more people			only 1 person	0.082	0.171	0.233	0.629	1.086 (0.777-1.518)
			2 people	0.347	0.111	9.804	0.002	1.415 (1.139-1.759)
**Where the dog is kept**	3.711	0.156						
reference category: only outdoors			only indoors	0.033	0.332	0.010	0.920	1.034 (0.540-1.982)
			in- and outdoors	0.274	0.345	0.633	0.426	1.316 (0.669-2.586)
**Hours spend walking the dog**	12.441	0.002						
reference category: > 3 hours			< 1 hour	0.725	0.230	9.895	0.002	2.065 (1.314-3.244)
			1-3 hours	0.118	0.165	0.516	0.472	1.126 (0.815-1.554)
**Allow the dog into the bed**	19.312	< 0.001						
reference category: yes			no	-0.483	0.110	19.312	< 0.001	0.617 (0.498-0.765)

Not coming back

**Global effect**			**Pairwise comparisons**					
**Factor**	**Wald χ^2**	**P value**	**Categories**	**B**	**SE**	**Wald χ^2**	**P value**	**Exp(B) (95% Conf Int)**
**Dogs' age**	26.449	< 0.001	-	-0.077	0.015	26.449	< 0.001	0.926 (0.900-0.954)
**Dogs' weight**	12.517	< 0.001	-	-0.011	0.003	12.517	< 0.001	0.989 (0.984-0.995)
**Weight-residual of height**	9.380	0.002	-	-0.156	0.051	9.380	0.002	0.855 (0.774-0.945)
**Neuter status**	1.778	0.182						
reference category: neutered			intact	0.119	0.090	1.778	0.182	1.127 (0.945-1.343)
**Age at acquisition**	2.392	0.495						
reference category: > 1 year			bred by owner	-0.418	0.304	1.896	0.169	0.658 (0.363-1.194)
			2–12 weeks	-0.013	0.126	0.010	0.920	0.987 (0.771-1.264)
			3-12 months	0.039	0.148	0.069	0.793	1.040 (0.778-1.388)
**Training experience**	56.405	< 0.001						
reference category: 3 or more types			no training	0.753	0.120	39.536	< 0.001	2.123 (1.679-2.685)
			1 type	0.852	0.127	44.922	< 0.001	2.345 (1.828-3.009)
			2 types	0.721	0.127	32.161	< 0.001	2.056 (1.603-2.638)
**Owners' gender**	1.259	0.262						
reference category: woman			man	0.130	0.116	1.259	0.262	1.139 (0.908-1.428)
**Owners' age**	25.621	< 0.001						
reference category: > 60 years			≤ 18 years	0.928	0.354	6.870	0.009	2.531 (1.264-5.067)
			19-30 years	0.260	0.280	0.862	0.353	1.297 (0.749-2.244)
			31-60 years	-0.060	0.273	0.048	0.826	0.942 (0.552-1.608)

**N of previous dogs**	44.259	< 0.001						
reference category: 3 or more dogs			no previous dog	0.774	0.126	37.719	< 0.001	2.168 (1.693-2.775)
			1 dog	0.695	0.136	26.075	< 0.001	2.003 (1.534-2.615)
			2 dogs	0.352	0.150	5.470	0.019	1.422 (1.059-1.909)
**Purpose of keeping the dog**	6.918	0.031						
reference category: not family member			family member only	-0.063	0.158	0.159	0.690	0.939 (0.689-1.279)
			family member+other	-0.272	0.157	3.003	0.083	0.762 (0.560-1.036)
**N of people in the household**	4.892	0.087						
reference category: 3 or more people			only 1 person	-0.231	0.142	2.644	0.104	0.794 (0.601-1.049)
			2 people	-0.176	0.090	3.857	0.050	0.839 (0.703-1.000)
**Where the dog is kept**	0.546	0.761						
reference category: only outdoors			only indoors	-0.203	0.280	0.527	0.468	0.816 (0.472-1.412)
			in- and outdoors	-0.183	0.288	0.404	0.525	0.833 (0.473-1.464)
**Hours spend walking the dog**	18.698	< 0.001						
reference category: > 3 hours			< 1 hour	0.741	0.178	17.414	< 0.001	2.098 (1.481-2.972)
			1-3 hours	0.285	0.139	4.213	0.040	1.330 (1.013-1.746)
**Allow the dog into the bed**	0.003	0.954						
reference category: yes			no	-0.005	0.085	0.003	0.954	0.995 (0.842-1.176)
